# Feasibility and acceptability of self-directed, remote dim-light melatonin onset collection in pediatric patients diagnosed with chronic pain

**DOI:** 10.3389/frsle.2025.1593196

**Published:** 2025-07-10

**Authors:** Nicole Tacugue, Jessica J. Love, Jakob M. Cherry, Jacqueline Lane, Joe Kossowsky

**Affiliations:** 1 Laboratory for Digital Assessment, Research, and Treatment, Department of Anesthesiology, Critical Care, and Pain Medicine, Boston Children’s Hospital, Boston, MA, United States; 2 The Lane Laboratory, Division of Sleep and Circadian Disorders, Harvard Medical School, Brigham and Women’s Hospital, Boston, MA, United States; 3 Department of Psychiatry, School of Medicine and Public Health, University of Wisconsin-Madison, Madison, WI, United States; 4 Department of Psychology, University of Wisconsin-Madison, Madison, WI, United States; 5 Program in Medical and Population Genetics, Broad Institute, Cambridge, MA, United States; 6 Center for Genomic Medicine, Massachusetts General Hospital, Boston, MA, United States; 7 Harvard Medical School, Boston, MA, United States

**Keywords:** pediatric, melatonin, chronic pain, circadian, DLMO

## Abstract

**Introduction:**

Sleep dysregulation is highly prevalent in pediatric chronic pain conditions and associated with poorer clinical outcomes. Interactions between underlying circadian misalignments and pain in pediatric populations remain unclear. Dim-light melatonin onset collections conducted in external lab settings are standard for measuring circadian rhythmicity by examining fluctuations in melatonin levels. However, present limitations prevent us from capturing a typical night’s sleep and minimize accessibility to broader populations due to geographic, financial, and temporal barriers. We investigated a novel approach in which participants complete collections in an entirely self-directed manner using an at-home diagnostic kit.

**Methods:**

Participants included pediatric patients with diagnosed chronic pain and healthy controls. The 3-week protocol involved sleep, activity, and light tracking, self-reported sleep diaries, a survey determining morningness-eveningness chronotypes, one self-directed home dim-light melatonin onset collection with objective compliance measures, and assessment of study protocol acceptability.

**Results and discussion:**

In a sample of pediatric patients with diagnosed chronic pain (*N* = 6, M_age_ =14.5, SD = 2.74, 66.7% female) and a subset of healthy controls (*N* = 6, M_age_ =13.3, SD=2.73, 50% female), both the Hockeystick method and 3 pg/ml dim-light melatonin onset threshold were employed to calculate salivary dim-light melatonin onset times in 8 of the 12 participants. On average, dim-light melatonin onset times were 1 h and 43 min earlier than self-reported sleep onset times. Our results illustrate the feasibility and accuracy of self-directed, remote dim-light melatonin onset collections in pediatric populations. With supplementary research validating this optimized approach to measure endogenous circadian phase, more specific aspects of sleep can be targeted in pain intervention strategies to further optimize clinical outcomes in a greater population of pediatric patients.

## 1 Introduction

Sleep in adolescence is characterized by major changes in sleep behavior regulation and circadian clock processes (Albinni et al., 2023). A common finding in pediatric sleep is the prominence of misaligned circadian rhythms (Christensen et al., 2019), which tend to occur in response to external environmental factors such as later bedtimes and early school schedules (Lauderdale et al., 2008). In pediatric populations, sleep dysregulation is associated with new onset chronic pain and exacerbation of pre-existing pain (Badawy et al., 2019). Comorbid sleep disturbance and chronic pain in pediatric patients are also associated with lower quality of life, greater emotional distress, and limitations in physical and cognitive functioning (Valrie et al., 2013). Without proper treatment, youth are at heightened risks of developing sleep disorders and chronic pain disorders that persist into adulthood (Palermo, 2020).

The extent to which circadian rhythms influence pain in the pediatric population remains unclear. Circadian rhythms regulate an individual’s chronotype, which is used to describe interpersonal differences in natural sleep-wake cycles and activity preferences across the 24-h day. Chronotypes typically shift throughout the lifespan, as they are heavily influenced by biological (Fischer et al., 2017), environmental (Crowley et al., 2015), and lifestyle (Haldar et al., 2021) factors. Younger children often exhibit morning chronotypes as their circadian systems rapidly develop, tending to wake earlier and be most active in the morning. Youth commonly shift toward an evening chronotype as a result of pubertal changes delaying melatonin release to promote sleep, and thus prefer later bedtimes and nighttime activity (Karan et al., 2021). Self-report questionnaires, such as the Morningness-Eveningness Questionnaire (MEQ) (Horne and Ostberg, 1976; Tonetti, 2007), are typically administered to determine circadian chronotype. The reported frequency of poor sleep in pediatric patients with chronic pain warrants more detailed consideration of chronotypes to better establish interventions tailored to individual circadian preferences.

Self-report inventories are frequently employed to measure sleep and activity in pediatric patients but are limited by inaccuracies and bias as well as the inability to measure underlying physiology (Lauderdale et al., 2008). More objective methods of sleep and activity assessment such as actigraphy devices provide a means of non-invasive and long-term data collection in free-living environments. These devices, however, are limited in their ability to produce multidimensional datasets reflective of various domains of sleep as they exclusively capture motion-based activity (Meltzer et al., 2012). Further research is needed that uses objective measurements of endogenous circadian phase to improve sleep characterization within various pediatric populations.

Currently, the gold standard method to assess circadian biorhythmicity is through completion of Dim Light Melatonin Onset (DLMO) collections, which capture internal melatonin production through saliva, blood, or urine samples (Danilenko et al., 2014). Patterns of melatonin concentration fluctuations are indicative of individuals’ circadian clock and can be used for diagnostic purposes of circadian rhythm disorders. Typically, DLMO collections are conducted in controlled laboratory environments within sleep or circadian clinics to account for requirements of dim-lighting and hourly salivary sample collections for 8 h in the evening (Benloucif et al., 2008). These conditions present geographic, financial, and temporal barriers that may compromise optimization of care.

One prior study has examined the applicability of in-home salivary collection in pediatric patients with craniopharyngioma but were limited by subjective reports of protocol adherence (Mandrell et al., 2018). Recent studies have explored an unconventional approach in which DLMO collections were completed fully remotely in adult populations with circadian rhythm disorders and produced results similar to standardly collected DLMOs (Bormes et al., 2023). No studies have yet examined remote DLMO collections in a pediatric population with chronic pain. We aim to determine the practicality of such a self-directed and fully remote approach in pediatric patients diagnosed with chronic pain used in conjunction with objective compliance measures. We hypothesize that this approach may represent an alternative means for better characterizing sleep in pediatric pain through measurements of melatonin concentration that otherwise might only be obtained using burdensome conventional methods associated with external sleep clinics. The current study, therefore, investigates the feasibility and acceptability of employing a self-directed approach to DLMO collections in a pediatric pain population.

## 2 Materials and methods

### 2.1 Participants

Participants included six patients actively seeking treatment for chronic pain (*N* = 6, mean age = 14.5, SD = 2.7, 4/6 (66.7%) female) and six healthy controls (*N* = 6, mean age = 13.3, SD = 2.7, 3/6 (50%) female). Those with chronic pain were recruited from a tertiary clinic specializing in pediatric pain management services located in the Northeast United States. Healthy control participants were recruited using pamphlets posted within the hospital and online social media platforms. Recruitment occurred between June 2024 and August 2024. IRB approval was received from Boston Children’s Hospital (IRB-P00047294) to conduct the study.

Participants were eligible for the study if they were (1) between 8 and 19 years of age, (2) able to wear an actigraphy watch, and (3) able to comprehend instructions in English. Participants were excluded if they (1) had a history of traumatic brain injury or stroke (Shekleton et al., 2010), or (2) were diagnosed with severe cognitive impairment (Naismith et al., 2014), seizure disorders (Paprocka et al., 2018), or dental conditions of gingivitis, xerostomia, or periodontitis.

In the chronic pain sample, eligible participants were identified through weekly screenings of clinic schedules conducted by a study team member. In the control sample, prospective participants scanned a QR code from the study flier directing them to a REDCap (Harris et al., 2009) questionnaire in which they provided their contact information. Participants in each group were contacted by a member of the study team via mail, email, or secure patient portal message with information regarding the study. A member of the study team contacted participants by phone several days after the initial message to further relay study details, address questions, and gauge interest in participation. Those who verbalized interest in participation were consented remotely and onboarded for study participation upon electronic receival of written informed patient assent and parental consent.

Consented participants were subsequently enrolled into the secure online study portal, Studytrax (Sciencetrax, 2021) to provide a dashboard for all study instruments and due tasks. Additionally, participants received a shipment of the at-home DLMO kit with all materials required for the protocol. Demographic data was collected from the Health and Lifestyle Questionnaire (Supplementary material: Health and Lifestyle Questionnaire) completed by participants at the start of the study. Participants additionally filled out the validated MEQ to assess their chronotypes. Upon completion of both instruments, they were granted access on the portal to begin sleep logging on a daily basis and schedule their DLMO collection date. DLMO collections were 8 hours in duration, beginning 6 hours before their average bedtime as calculated from sleep diary data, and ending 2 hours after their average bedtime. Participants were able to choose a DLMO date during the final week of the study convenient for their schedules, excluding Monday due to potential weekend social jetlag. Collected DLMO samples were returned to the research team under ideal temperatures using shipping materials included in the kit.Upon completion of the study, participants answered semi-structured interview questions online assessing feasibility and acceptability of the DLMO protocol.

### 2.2 At-Home DLMO kit

Enrolled participants were provided with a study kit containing all materials necessary to complete their remote DLMO either by mail or in person. Each kit included an instruction manual (Supplementary material: At-Home Kit Instructions), ActTrust 2 actigraphy watch (Danilenko et al., 2022), VWR Digital Luxmeter LXM001 light meter, blue light-blocking glasses, nine untreated Sarstedt Salivettes (Starstedt, Germany) enclosed in a bottle, a medication event monitoring system (Hartman et al., 2019) (MEMs) bottle cap to record exact timings of each collected sample, a temperature sensor, a freezer bag with ice packs to store samples, and a prepaid shipping label for kit return. If participants wished to use their laptops during the collection period, they were instructed to wear the blue light-blocking glasses, adjust their screens to the dimmest setting, and place devices at least 5 feet away. Blue light-blocking glasses provided an additional, protective measure to ensure that the results of any light exposure were being dampened. Miscellaneous items were included for ancillary purposes, such as a marker, toothbrush, 18 tealights, 2 extra salivettes, and contractor-grade trash bags to prepare the collection space (Supplementary material: At-Home Kit Contents). The toothbrush allowed participants to brush their teeth without toothpaste if they had eaten prior to sample collections in order to avoid affecting melatonin production. Tealights were specifically tested by our study team, ensuring they did not produce enough lux to suppress melatonin production. contractor-grade trash bags were provided for those without blackout curtains to avoid external light from entering the environment and increasing lux exposure. Participants were asked to place the ice packs in their freezer upon arrival of the kit. These ice packs, along with the freezer bag, were only used for the return shipment. Participants were asked to keep saliva samples in their freezer, stored away in biohazard bags that were provided, until the end of the study.

### 2.3 Data collection

#### 2.3.1 DLMO collection timing

We calculated the timeframe of the DLMO 24 hours prior to the scheduled DLMO date. The last 2 self-reported sleep log entries of time asleep prior to the DLMO were averaged to calculate a habitual bedtime, which was then rounded to the nearest hour. The DLMO sampling window was set to start 6 h before the approximated habitual bedtime to 2 h after. Retrospective calculations were conducted to compare DLMO sampling windows when averaging the prior 7 sleep log entries of time asleep to when averaging the prior 2 sleep log entries of time asleep.

#### 2.3.2 DLMO collection

One salivary DLMO collection was completed during each participant’s final week of the study. Research staff instructed participants to independently complete DLMO collection protocols without parental involvement, only using assistance from resources provided by the study. Prior to collection, participants reviewed instructional materials to prepare their environment to adhere to dim light conditions under 10 lux. Participants were instructed to hold the light meter at eye level and report the lux level of their dim light environment to the study team 30 min before collecting the first sample. Participants maintained a resting position for the duration of the collection period. Automatic reminders from research staff were sent to participants’ mobile devices to ensure samples were collected at designated times. Hourly samples were obtained for a total of 9 saliva samples in the evening, approximating 8 hours per collection. The first 9 participants collected saliva via a cotton swab placed sublingually for 3 min. Following completion of the final sample, all salivettes remained under freezing conditions in the participant’s freezer. At the end of the study, participants were instructed to place samples and ice packs in the provided freezer bag, which was sealed for optimal travel. To address inconsistencies in salivette labeling and sample volume, two protocol changes were incorporated for the final 3 participants. Research staff pre-labeled salivettes according to sample number and requested participants to separately label numbers on salivettes by the order in which samples were collected to ensure the correct order of salivettes was employed. These participants were alternatively asked to chew a cotton swab for 3 min to better stimulate saliva production.

#### 2.3.3 ActTrust 2 watch

The ActTrust 2 actigraphy watch was worn on all participants’ non-dominant wrists at the beginning of the second week in the study. Participants were instructed to press the button on the watch face to indicate each time they had gone to bed and each time they had woken up. The ActTrust 2 contains multiple sensors capable of tracking data related to light exposure, physical activity, temperature fluctuations, and sleep/wake times (Danilenko et al., 2022).

#### 2.3.4 Morningness-eveningness chronotype

Chronotypes were characterized using a self-report inventory measuring individual differences on a 5-point scale reflective of how alert or active respondents are at various times throughout the day (1 = minimal morning preference; 5=maximal morning preference). A validated abridged 10-item version of the Morningness-Eveningness Questionnaire adapted for pediatric participants (Tonetti, 2007) (rMEQ-A) was administered for participants under the age of 18. Participants 18 years of age and older received a corresponding adult version of the assessment (Horne and Ostberg, 1976). Raw scores were summed to a total score (10–43). Higher scores of 28–43 indicate greater preferences toward morningness, lower scores of 10–20 indicate greater preferences toward eveningness, and scores between 21 and 27 are categorized as neither.

#### 2.3.5 Feasibility and acceptability

A series of compliance measures were gathered to determine feasibility of the DLMO collection, as indicated by validity of samples. DLMO compliance measurements included the following: completion of both Pre- and Post-Collection Attestations on the patient portal, consistency of light levels less than 10 lux throughout the collection, and samples obtained within 5 min of the scheduled collection time. Samples were considered valid if a participant met all conditions of compliance measures.

Participant compliance with daily sleep logs and actigraphy wear were examined to further assess feasibility of the study. Compliance rates for sleep logging were calculated for each participant within both samples, computed from the number of days in which sleep logs were submitted out of the total number of days in the study. Compliance rates for actigraphy wear were calculated for each participant within both samples. If the participant did not remove the actigraphy watch throughout the night, then the day was considered compliant. Computations were calculated based on the number of compliant days out of the total number of days in the study.

Acceptability was evaluated using semi-structured interview questions to survey patient engagement, ease of instruction comprehension, and DLMO competency. Participants were asked to endorse (Yes/No) whether they deviated from protocol at any point in the study through a variety of specific scenarios (e.g., “*Did you consume any caffeine in the 24 hours prior to completing your DLMO collection*?”). Moreover, participants rated the degree of confidence in their ability to prepare for and collect salivary samples on a 5-point scale (e.g., “*I felt confident completing the DLMO collection from the written instructions alone*.” 1=*strongly disagree*, 5=*strongly agree*). To conclude the semi-structured interview, participants were asked open-ended questions to obtain feedback on the resources provided, context for any encountered challenges, and suggestions for future improvements.

### 2.4 Data processing and analysis

#### 2.4.1 Self-report questionnaires

SPSS Version 28.0 (IBM Corp, 2021) was used for descriptive analyses of outputs from administered self-report questionnaires. We conducted Fisher’s Exact Test to examine differences in chronotypes andcharacteristics of health and lifestyle between the pain cohort and healthy controls. Fisher’s Exact Test was employed to avoid distributional assumptions given our small sample size. Statistical significance was determined at *p* < 0.05.

#### 2.4.2 DLMO collection samples

Upon receiving the returned kit, all DLMO samples were inspected to record qualitative temperature data [“minimal condensation” (little/no droplets or fogging in the tube), “moderate condensation” (few droplets and/or fogging), or “heavy condensation” (many droplets or liquid in the tube)]. Data was extracted from the actigraphy watch and tracker bottle caps using ActStudio (Condor Instruments, 2013) and MEMs Adherence Software (Aardex Group, 2016), respectively to objectively measure compliance. Differences in compliance rates between groups was determined using the Wilcoxon rank-sum test. Following inspection, samples were kept under freezing conditions at−80°C during shipment to SolidPhase, Inc. (Portland, Maine) for melatonin assay using the Novolytix Direct Saliva Melatonin RIA kit (ALPCO Diagnostics, Windham, New Hampshire). Intra- and inter-assay coefficients of variation were 7.9% and 9.8%, respectively.

#### 2.4.3 Sample-based DLMO timing calculation

Timing of DLMO was calculated by a study team member using the Hockeystick algorithm, which determines DLMO by both the threshold and hockeystick methods. The Hockeystick method was used to estimate the most likely location where the melatonin profile begins to rise, through the use of fitting the profile to a piecewise function (Danilenko et al., 2014; Crowley et al., 2016). Additionally, the 3 pg/ml threshold for determining DLMO using salivary melatonin has been suggested to be reported for comparison of DLMO results between studies, and as such was also included (Benloucif et al., 2008).

## 3 Results

**FIGURE 1:**
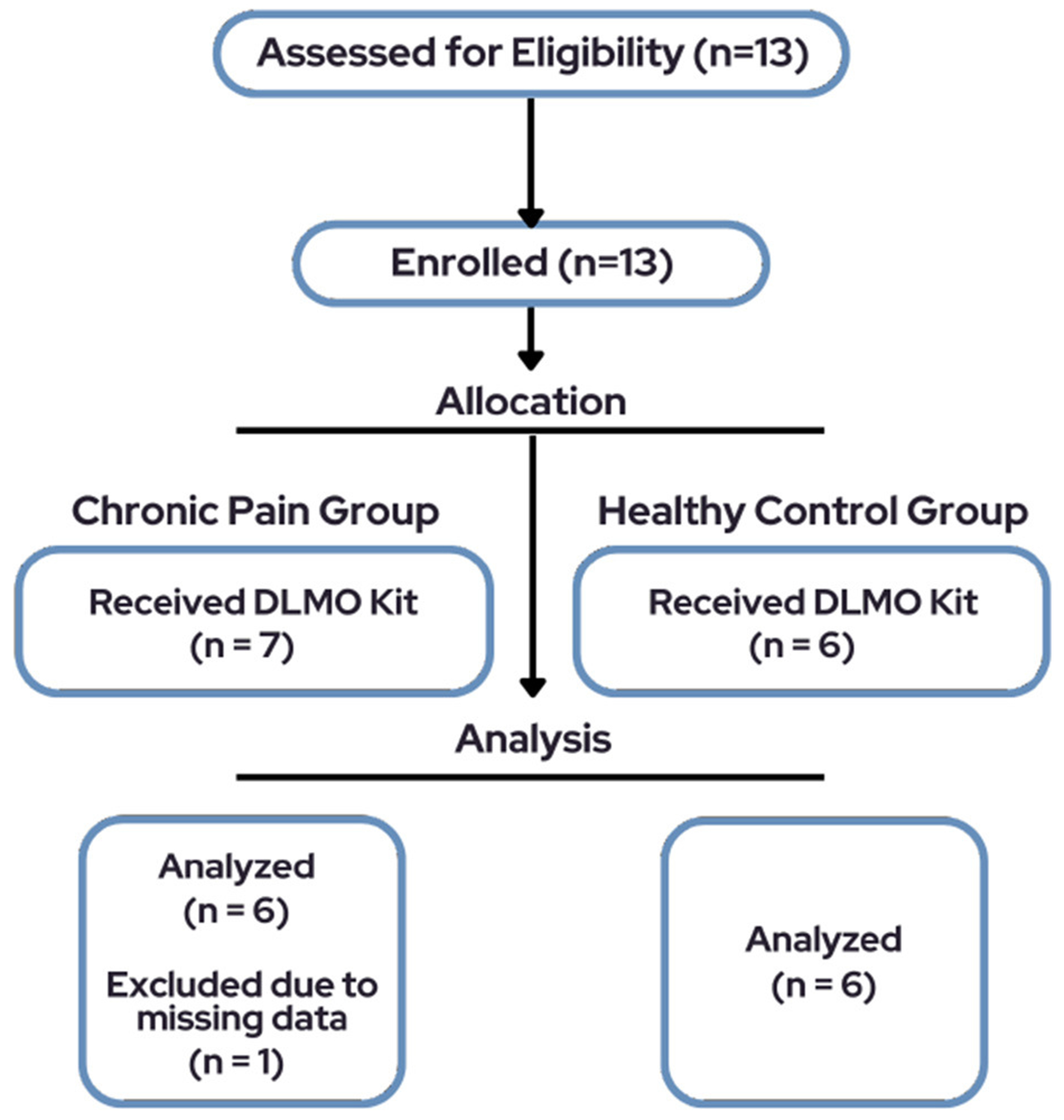
Participant flow diagram of the study.

The total sample consisted of 13 pediatric participants, however only 6 participants (66.7% female, M_age_ = 14.5, SD = 2.74, range = 10–19) diagnosed with chronic pain and a subset of six healthy controls (50% female, M_age_ = 13.3, SD = 2.73) were included in final analyses due to missing data for one participant (Figure 1). See Table 1 for demographic characteristics.

### 3.1 Health and lifestyle

No significant group differences were identified in health and lifestyle characteristics between the pain population and healthy controls. 66.6% of participants represented families living in a 3 or more person household. No participants indicated any household members under the age of 2. Regarding psychological phenotypes, 33.3% of participants self-reported diagnoses of depression, 66.7% self-reported anxiety, 16.7% self-reported obsessive compulsive disorder, 8.3% self-reported attention-deficit/hyperactive disorder, and 16.7% self-reported post-traumatic stress disorder. No participants reported clinical diagnoses of schizophrenia, bipolar affective disorder, dementia, narcolepsy, obstructive sleep apnea, or insomnia. See Table 2 for characteristics across both cohorts.

### 3.2 Morningness-eveningness chronotype

Findings from the rMEQ-A revealed a total of 4 participants with chronic pain (66.7%) and 4 healthy controls (66.7%) demonstrated a morningness preference with total scores between 28-43, and no participants demonstrated an eveningness preference with total scores between 10 and 20. Two participants with chronic pain (33.3%) and 2 healthy controls (33.3%) did not indicate a preference for either morningness or eveningness as their total scores were within the 21–27 range.

### 3.3 Compliance measures

Participants engaged remotely using an online portal and a mailed (*n* = 11) or physically provided (*n* = 1) kit with all materials for actigraphy and an at-home DLMO sample collection. Across the study period, participants demonstrated an overall compliance rate of 98.02% for daily sleep log completion, averaging 20.58 entries out of a total of 21.

All participants attested to refraining from caffeine and melatonin consumption 24 h before sample collection. Further, the entire cohort reported lux levels below 10 prior to initiating DLMO collection, confirming compliance with environmental requirements. Following DLMO completion, all participants confirmed samples were immediately placed in a freezer.

Validity of each salivette was determined based on data recorded from the MEMs cap used on the bottle which was opened to retrieve a cotton swab for each sample. Samples were considered valid if they were collected within 5 min of the expected time point. 10 of the 12 total participants (83%) had valid MEMs data, as 1 participant failed to return the MEMs cap, and 1 other participant did not close the cap fully between each sample collection, resulting in no times being recorded. As presented in Table 3, 42 of 54 samples (78%) in the control group and 44 of the 54 samples (81%) in the pain group were compliant with the sample collection within 5 min of the scheduled time during the DLMO collection. A Wilcoxon rank-sum test yielded a p-value of 1.00, indicating no statistically significant difference in compliance between the groups. Overall, a total of 86 of the 108 samples (80%) were deemed valid based on MEMs timing.

Lux levels were successfully recorded for all 12 DLMOs (sampling rate=25 Hz; down-sampled to 30-second-long epochs for analysis). Light level compliance was analyzed for approximately 8 hours and 30 min, starting 30 min before the first sample collection and ending at the collection of the final sample. Lux related compliance for an individual sample was determined from whether the 10 lux threshold was crossed in the time beginning from either the start of the DLMO or from the previous sample collection until the time of collection of the sample of focus. The 5th percentile of the 30-second epoch distribution of lux values was 0 across all 12 DLMOs, while the median lux value was 0 across 9 of the 12 DLMOs (75%), with one sample having a median of 0.8 lux, and the other two having a median lux of 0.09. The 95th percentile of the distribution of lux values was < 10 lux for 9 of the 12 DLMOs (75%), with 3 individuals having their 95th percentile lux as 97.15, 216.82, and 269.57 respectively. Each of these individuals had high light expressed starting from the 30 min before the first sample collection until sample 2 was collected, and remained compliant after that point. The most commonly non-compliant sample by light was sample 1, as only 3 out of the 12 initial samples (25%) were considered valid under the 10-lux threshold. As presented in Table 3, 33 of the 54 samples (61%) in the control group and 47 of the 54 samples (87%) in the pain group were compliant with the 10-lux threshold during the DLMO collection. A Wilcoxon rank-sum test yielded a p-value of 0.10, indicating no statistically significant difference in compliance between the groups. By the 10-lux threshold, 80 of the 108 samples (74%) were considered valid.

In regards to complete sample compliance by both the 10 lux and sample collection timing measures, 27 of the 54 samples (50%) in the control group and 38 of the 54 samples (70%) in the pain group were compliant. A Wilcoxon rank-sum test yielded a p-value of 0.36, indicating no statistically significant difference in compliance between the groups. Between both the 10 lux and sample collection timing measures, in total, 65 of the 108 (60%) of samples were considered valid. See Table 3 for complete compliance measurements of individual participants and overall group averages.

### 3.4 DLMO collection

Prior to collection, DLMO sampling window times were determined based on averages from participants’ previous 2 sleep logs. Retrospective calculations for DLMO sampling window times were conducted based on averages from their previous 7 sleep logs, and no significant differences were observed when compared to averages from their previous 2 sleep logs.

Using salivary melatonin data, we calculated DLMO times. Using both the Hockeystick method and a 3 pg/ml threshold, we successfully calculated DLMO times in 8 of the 12 participants. When using the 2SD method, we initially recorded 7 DLMO times, however, due to the possibility that the participant flipped the labeling of the samples, which led this 1 DLMO time to be considered invalid, so 6 were calculated. Overall, 8 of the 12 participants, 4 in the control group and 4 in the chronic pain group, had times successfully calculated. Table 4 demonstrates that DLMO times on average were 1 h and 43 min earlier than self-reported sleep times (Chronic Pain: 11:15 PM, Controls: 10:43 PM). When comparing DLMO times determined using a 3 pg/ml threshold between the control and pain groups, a Wilcoxon rank-sum test yielded a p-value of 0.11, indicating no statistically significant difference in DLMO times between the groups.

### 3.5 Feasibility and acceptability

Regarding feasibility of remote DLMO collection, 79.3% of participants endorsed confidence in their ability to complete the collection from written instructions alone, and a total of 77% of participants reported they had sufficient resources to complete DLMO collection independently. 100% of participants expressed confidence in preparing their environment prior to collection. 38.5% of participants verified use of contractor-grade trash bags and tape included in the kit to block out surrounding light, whereas the remainder of participants utilized personal black out curtains or blinds.

Relative to acceptability of other required study tasks, 78% of participants considered daily sleep log entries as either very or extremely easy, and 22% of participants considered daily sleep log entries as only slightly easy. 76.9% of participants reported zero problems wearing the actigraphy watch throughout the study. Issues encountered with the device were limited to unanticipated battery depletion and temporary rash from irritation.

Free response answers were collected to assess the most difficult aspects of the study. Common themes of difficulty in participant comments included remembering to push the event-button on the actigraphy watch to indicate bedtime and wake time, and the restrictions in activity, eating, and sleeping for the duration of the DLMO collection.

Participants were requested to offer feedback to incorporate future protocol improvements. Common themes suggested for enhancing protocols involved sending more reminders for study-specific tasks (*n* = 4), further expanding food options acceptable during the DLMO collection (*n* = 2), and increasing clarity in the instruction packet (*n* = 2). For improving study task adherence, a large majority of participants (*n* = 7) proposed to incorporate text message reminders for completing daily sleep logs and pressing the event-button on the actigraphy device for bedtime and wake time. Specific to refining the instruction packet, participants recommended for research staff to condense the wording as well as include more picture-based guidant instructions (*n* = 4). 50% of participants had no suggestions for additional materials to include in the kit for future protocols. Other participants advised research staff to identify more effective tape for environment preparation, include more pain medication, and provide a list of acceptable food items separate from the instruction guide.

## 4 Discussion

To the authors’ knowledge, the current study represents the first attempt to implement self-directed, remote DLMO collection in the pediatric pain population. Protocols from the Circadia Pilot Study (Bormes et al., 2023), originally conducted in adults, were adapted to accommodate youth. Given our specific focus on feasibility and acceptability of at-home DLMO collection itself, modifications involved minimizing participant burden by omitting options to conduct a blood draw or DNA collection, reducing study requirements from 2 DLMO collections to 1, and shortening study duration from 5 to 6 weeks to 3 weeks. Other modifications involved using an alternative MEQ version validated for the pediatric population, integrating semantic adjustments to the Health and Lifestyle questionnaire to accommodate youth, and including a final assessment to acquire feedback on overall experience.

Objective compliance measures examined our participants’ ability to adhere to study protocols using the provided resources: the kit consisted of DLMO-related supplies, written instructional guides, and remote access to research staff for remaining questions throughout the collection period. Validity of saliva samples were calculated based on standard DLMO thresholds of light exposure and sample timing using Actiwatch and MEMs cap data, respectively (Molina and Burgess, 2011). Our findings in both the chronic pain cohort and healthy controls were overall consistent with DLMO results produced from traditional collections completed in external labs under staff supervision (Danilenko et al., 2014).

Contrary to commonly observed shifts toward evening preferences in the adolescent population (Karan et al., 2021), our participants presented with MEQ scores only representative of morning chronotypes or no preference for either chronotype. Based on population-level data indicating a 19% prevalence of evening chronotypes in adolescents, there remains a small, yet statistically plausible, 8% probability of recruiting zero evening chronotypes in our sample of twelve. This absence may also reflect sample characteristics: our cohort consisted of both younger and older adolescents, with younger adolescents tending to be more morning-oriented (Randler et al., 2017), and a predominance of females, who generally exhibit greater morningness than males (Randler, 2011). In addition, evening preferences may have been underreported due to external influences on sleep behavior. Parental enforcement of earlier bedtimes or scheduling demands from school or clinical care can lead adolescents to report sleep-wake behaviors consistent with morning chronotypes, even if their intrinsic preferences differ. Further, youth with chronic pain may adhere to behavioral or pharmacologic regimens promoting earlier sleep timing for pain management (Palermo et al., 2022), potentially masking underlying evening chronotypes.

In our prior remote DLMO pilot study in healthy adults and individuals with Delayed or Advanced Sleep Wake Phase Disorder, we successfully calculated a DLMO time in 14 of the 20 collections (70%) (Bormes et al., 2023). In the current study’s pediatric population, we successfully calculated a DLMO time in 8 of the 12 collections (66.7%). Using a Fisher’s Exact Test, a *p*-value of 0.61 was found, indicating no significant difference in the success rate between the two groups.

### 4.1 Limitations

Given the exploratory nature of the study, results may not be generalizable to a broader population due to our small sample size and lack of racial diversity Our sample may not fully represent the diversity of chronotypes and sleep behaviors found within broader pediatric populations. Non-significant findings should be interpreted with caution, as they may be indicative of insufficient power considering sample size. Although our sample size limits generalizability, our findings provide preliminary insight into the feasibility and acceptability of self-directed DLMO collection in the pediatric population, thus serving as a framework for future larger-scale studies with more demographically diverse samples.

Despite successful DLMO findings, several unforeseen challenges arose presumably as a result of the novel framework being trialed in a new patient population. Melatonin assays from the initial batch of samples produced inconsistent results, as 3 participants did not provide enough quantities of saliva to compute calculations. Other issues from these initial participants involved 1 not fully securing the MEMs cap after each saliva sample, 1 failing to number some samples according to the order they were collected, and 1 only wearing the actigraphy watch on the day of DLMO collection in addition to misplacing the MEMs cap.

To address such issues, we incorporated protocol changes for the final three participants: (1) participants were instructed to chew on collection swabs rather than keep them stagnantly sublingual in order to stimulate saliva production for sufficient salivary volume, (2) research staff pre-labeled salivettes with numbers and requested for participants to separately mark salivettes with numbers to ensure they were collected in the proper chronological order, and (3) participants received additional reminders to completely close the MEMs cap after each sample. Results from 2 of these 3 participants were able to successfully identify DLMO profiles, however the remaining participant appeared to re-label their salivettes in opposite chronological order based on their results, indicating a potential caveat to pre-labeling tubes. Future studies should consider other youth-friendly methods such as color-coding salivettes to ensure samples are obtained in correct sequential order (Mandrell et al., 2018). Given that DLMO times were successfully calculated for the majority of participants prior to introducing protocol changes, these initial inconsistencies were most likely reflective of individual user error.

We acknowledge a potential limitation in our employed methods for determining collection period timing as we instead averaged 2 sleep log entries of time asleep for calculations. Current literature tends to examine around 7 self-reported sleep log entries of time asleep to calculate timing for the DLMO collection period (Murray et al., 2021). However, retrospective calculations using 7 sleep log entries of time asleep revealed no significant differences in sleep timing compared to calculations using 2 sleep log entries of time asleep that would prevent DLMO identification. Our pilot data demonstrates the feasibility of capturing DLMO timing based on less sleep log entries, thus signifying a possible avenue for future studies to further reduce participant burden by minimizing sleep log requirements.

Although participants reported abstaining from intake of caffeine and melatonin supplements 24 hours prior to DLMO collection, a further consideration is that the study did not account for potential confounders such as habitual usage of such supplements and their effects on circadian timing. Regular caffeine consumption may interrupt endogenous melatonin production, potentially delaying DLMO timing (Reichert et al., 2021), whereas supplemental melatonin intake may cause advancements or delays in circadian phase depending on habitual timing of consumption (Zwart et al., 2018). Similarly, future studies should additionally consider the implications of psychological comorbidities that may also cause delays in DLMO timing (Sivertsen et al., 2015).

Future research should strive to examine feasibility and acceptability of remote DLMO collection in a larger and more diverse sample of circadian profiles within the pediatric population. Finally, in considering participant feedback, we recommend incorporating further adjustments to future protocols to optimize user experience and data quality. Key recommendations involve shifting toward more visual-based instructional guidance for increased tolerance in youth, as well as introducing alert notifications for sleep logs and actigraphy event-marking. Our data supports the potential for self-directed remote DLMO collection to be a low-burden alternative to in-lab measures of sleep characterization, especially for pediatric patients with chronic pain experiencing accessibility-related barriers. However, considering our small sample size and requirements for protocol improvements, we recommend further validating this method and standardizing protocols in larger cohorts with greater diversity prior to implementing self-directed remote DLMO collections on a more extensive scale.

### 4.2 Conclusions

Findings from the current study suggest overall feasibility, acceptability, and accuracy of self-directed, at-home DLMO collection in the pediatric population. Our proposed remote approach demonstrates notable advantages over conventional DLMO collection protocols, as it offers ease of enrollment and bypasses requirements to conduct serial saliva sampling in an external lab under supervision while producing similar results to standard practices, thus decreasing healthcare barriers and increasing accessibility to circadian phase assessment. Considering the high prevalence of sleep disturbance in youth (Lewien et al., 2021) in general but most particularly in those with chronic pain (Palermo et al., 2012), further research incorporating circadian rhythm assessments with minimal burden is necessary to better characterize circadian disruptions during the period of adolescence in which sleep is especially vulnerable to dysfunction.

## Supplementary Material

Table 1

Data Sheet 3

Data Sheet 2

Data Sheet 1

The Supplementary Material for this article can be found online at: https://www.frontiersin.org/articles/10.3389/frsle.2025.1593196/full#supplementary-material

## Figures and Tables

**TABLE 1:** Demographics.

Variable	Total	Pain	Control
	*N* = 12	*n* = 6	*n* = 6
Mean age	13.9 (2.7)	14.5 (2.7)	13.3 (2.7)
**Race**			
White or European American	10 (83.3)	4 (66.7)	6 (100)
Asian	1 (8.3)	1 (16.7)	0 (0)
Other/multiracial	1 (8.3)	1 (16.7)	0 (0)
**Gender**			
Female	7 (58.5)	4 (66.6)	3 (50)
Male	5 (41.7)	2 (33.3)	3 (50)

**TABLE 2:** Self-reported diagnoses.

Diagnoses, *n* (%)	Total	Pain	Control
	*N* = 12	*n* = 6	*n* = 6
Depression	4 (33.3)	2 (33.3)	2 (33.3)
Anxiety	8 (66.7)	5 (83.3)	3 (50)
Bipolar disorder	0 (0)	0 (0)	0 (0)
Dementia	0 (0)	0 (0)	0 (0)
ADHD	1 (8.3)	1 (16.7)	0 (0)
Schizophrenia	0 (0)	0 (0)	0 (0)
OCD	2 (16.7)	1 (16.7)	1 (16.7)
Autism	1 (8.3)	0 (0)	1 (16.7)
PTSD	2 (16.7)	1 (16.7)	1 (16.7)
Restless leg syndrome	1 (8.3)	1 (16.7)	0 (0)
Narcolepsy	0 (0)	0 (0)	0 (0)
Obstructive sleep apnea	0 (0)	0 (0)	0 (0)
Insomnia	0 (0)	0 (0)	0 (0)
Other	0 (0)	0 (0)	0 (0)

ADHD, attention-deficit/hyperactivity disorder; OCD, obsessive compulsive disorder; PTSD, post-traumatic stress disorder.

**TABLE 3:** Compliance of samples during DLMO collection.

Participant	Maximum of 10 lux environment maintained	MEMs time recorded within 5 min of scheduled time	Fully compliant samples
Control 1	3/9 (33%)	0/9 (0%)	0/9 (0%)
Control 2	1/9 (11%)	9/9 (100%)	1/9 (11%)
Control 3	8/9 (89%)	9/9 (100%)	8/9 (89%)
Control 4	7/9 (78%)	6/9 (67%)	4/9 (44%)
Control 5	7/9 (78%)	9/9 (100%)	7/9 (78%)
Control 6	7/9 (78%)	9/9 (100%)	7/9 (78%)
Pain 1	7/9 (78%)	9/9 (100%)	7/9 (78%)
Pain 2	7/9 (78%)	8/9 (89%)	7/9 (78%)
Pain 3	9/9 (100%)	0/9 (0%)	0/9 (0%)
Pain 4	9/9 (100%)	9/9 (100%)	9/9 (100%)
Pain 5	7/9 (78%)	9/9 (100%)	7/9 (78%)
Pain 6	8/9 (89%)	9/9 (100%)	8/9 (89%)
**Group averages**			
Control group	33/54 (61%)	42/54 (78%)	27/54 (50%)
Pain group	47/54 (87%)	44/54 (81%)	38/54 (70%)
*Overall*	80/108 (74%)	86/108 (80%)	65/108 (60%)

DLMO, dim light melatonin onset; MEMs, medication event monitoring system bottle caps.

**TABLE 4:** DLMO and bedtime calculations.

Outcome measure	Total	Pain	Control
	N = 12	n = 6	n = 6
**DLMO**			
DLMO calculation rate (%)	8/12 (66.7%)	4/6 (66.7%)	4/6 (66.7%)
Average DLMO by 3 pg/mL threshold in decimal hours (SD)	21.16 (1.58)	22.18 (1.27)	20.14 (1.08)
**Bedtime**			
Average bedtime in decimal hours with successful DLMO (SD)	22.98 (0.48)	23.25 (0.27)	22.71 (0.51)

DLMO, dim light melatonin onset.

## Data Availability

The raw data supporting the conclusions of this article will be made available by the authors, without undue reservation.
