# Supplementary material for: Feasibility and acceptability of self-directed, remote dim-light melatonin onset collection in pediatric patients diagnosed with chronic pain
Source: Front Sleep. 2025 Jul 10;4:1593196. doi: 10.3389/frsle.2025.1593196 (PMC12435390; doi:10.3389/frsle.2025.1593196)
Supplement: Supplementary file 1 [file Table_1.docx]

| *Supplementary Table 1. Medication and Supplement Use Characteristics* | | | | |
| --- | --- | --- | --- | --- |
|  | **Total** | **Pain** | **Control** |  |
|  | N = 12 | n = 6 | n = 6 |  |
| **Reported Substances, n (%)** |  |  |  | |
| Caffeine | 6 (50) | 4 (66.7) | 2 (33.3) | |
| Melatonin | 2 (16.7) | 1 (16.7) | 1 (16.7) | |
| NSAIDs | 2 (16.7) | 2 (33.3) | 0 (0) | |
| Birth Control | 2 (16.7) | 2 (33.3) | 0 (0) | |
| Methylphenidate | 1 (8.3) | 1 (16.7) | 0 (0) | |
| Lorazepam | 1 (8.3) | 1 (16.7) | 0 (0) | |
| Antihistamines | 1 (8.3) | 1 (16.7) | 0 (0) | |
| Other | 2 (16.7) | 2 (33.3) | 0 (0) | |
|  |  |  |  | |
| *Abbreviations.* NSAIDs, nonsteroidal anti-inflammatory drugs. | | | | |
