# Supplementary material for: Feasibility and acceptability of self-directed, remote dim-light melatonin onset collection in pediatric patients diagnosed with chronic pain
Source: Front Sleep. 2025 Jul 10;4:1593196. doi: 10.3389/frsle.2025.1593196 (PMC12435390; doi:10.3389/frsle.2025.1593196)
Supplement: Supplementary file 2 [file Data_Sheet_1.docx]

**Supplementary Material: Health and Lifestyle Questionnaire**

| 1. What is your date of birth? | (MM-DD-YYYY) |
| --- | --- |
| 1. What is/was your assigned sex at birth? | Male  Female  Intersex/Ambiguous |
| 1. What is your current gender identity? | Male/ Man  Female/ Woman  Nonbinary  Trans female/ trans woman  Trans male/ trans man  Gender queer or gender non conforming  Other  Decline to answer |
| 1. What are your pronouns? | She/Her/Hers  He/Him/His  They/Them/Theirs  Other (please specify) |
| 1. How tall are you? | _____Feet  _____Inches |
| 1. What is your estimated weight? | _____ Pounds |
| 1. What is your race/ethnic background? Select all that apply. | Indigenous American and/or Alaska Native  A person having origins in any of the original peoples of North and South America (including Central America) who maintains cultural identification through tribal affiliation or community attachment.  Asian  A person having origins in any of the original peoples of East Asia, Southeast Asia, or the Indian Subcontinent, including, for example, Cambodia, China, India, Japan, Korea, Malaysia, Pakistan, the Philippine Islands, Thailand, and Vietnam.  Black or African American  A person having origins in any of the black racial groups of Africa.  Hispanic or Latinx  A person of Latin American origin or descent, or a person of Spanish origin, regardless of race.  Native Hawaiian or Other Pacific Islander  A person having origins in any of the original peoples of Hawaii, Guam, Samoa, or other Pacific Islands.  Middle Eastern or North African  A person having origins in any of the original peoples of the Middle East, or North Africa.  White or European American  A person having origins in any of the original peoples of Europe.    Other (please specify)  Decline to Answer |
| 1. Please indicate if any of the following were completed: | Dim Light Melatonin Onset  Actigraphy/Wearable activity device  Sleep Log or Diary  None of the Above  Decline to Answer  Other (please specify) |
| 1. Not counting yourself, how many people do you live with in your household? | 0  1  2  3  4  5  6+ |
| 9a. (If anything over 0 selected) Thinking about your household, how many are under the age of 2? | 0  1  2  3  4  5  6+ |
| 1. Have you ever been diagnosed with one of the following psychiatric disorders? Please select all that apply. | Clinical Depression  Anxiety Disorder  Bipolar Affective Disorder  Dementia  Attention-deficit/hyperactivity Disorder  Schizophrenia  Obsessive Compulsive Disorder  Autism  Post-Traumatic Stress Disorder  Other: _____  None  Decline to Answer |
| 1. Have you ever been diagnosed with one of the following sleep disorders? Please select all that apply. | Restless Leg Syndrome  Narcolepsy  Obstructive Sleep Apnea  Insomnia  Other: _____  None  Decline to Answer |
| 1. Do you typically consume caffeinated beverages at least 1x a day? | Yes  No |
| 12a. As part of the study you will be required to withhold consuming caffeine for up to 48 hours for sample collection purposes. Please select an option regarding your caffeine consumption during the study collection: | Yes – I will be able to withhold consumption of caffeine for up to 48 hours for the duration of the study.  No – I will not be able to withhold consumption of caffeine for up to 48 hours for the duration of the study. |
| 1. Do you take melatonin to help you fall asleep and/or for other reasons? | Yes  No |
| 13a. As part of the study you will be required to withhold using melatonin for up to 48 hours for sample collection purposes. Please select an option regarding your melatonin use during the study collection: | Yes – I will be able to withhold use of melatonin for up to 48 hours for the duration of the study.  No – I will not be able to withhold use of melatonin for up to 48 hours for the duration of the study. |
| Please select all of the following medications that you used within the past month: | Over the Counter Nonsteroidal Anti-inflammatory drugs (NSAIDS)  Birth Control (pill, IUD, vaginal ring, implant, hormone shot, etc)  Lithium  Ramelteon  Provigil  Zolpidem (Ambien)  Zalepion (Sonata  Eszopicione (Lunesta)  Adderall  Dextroamphetamine (Dexidrine)  Methylphenidate (Ritalin, Concerta)  Metaprolol (Lopressor, Toprol XL)  Mexiletine (Mexitil)  Propranolo (Hemangeol, Inderal LA, Inderal XL, InnoPran XL, Inderal)  Verapamil (Calan, Isoptin SR, Verelan, Isoptin, Calan SR, Isoptin I.V., Covera-H-S, Verelan PM)  Flucortisone (Florinef)  Midodrine (Orvaten, ProAmatine)  Fluvoxamine  Luvox  Moxiflaxin  Delafloxacin  Ciproflaxin  Gemifloxacin  Levofloxacin  Ofloxacin  Diazepam (Valium)  Lorazepam (Ativan)  Alprazolam (Xanax)  Coedine  Hydrocodone (Vicodin, Hycodan)  Morphine (MS Contin, Kadian)  Oxycodone (Oxycontin, Percoset)  Hydromorphone (Dilaudid)  Fentanyl (Duragesic)  Over the Counter Antihistamines  Other: _____  None  Decline to Answer |
