# Supplementary material for: Feasibility and acceptability of self-directed, remote dim-light melatonin onset collection in pediatric patients diagnosed with chronic pain
Source: Front Sleep. 2025 Jul 10;4:1593196. doi: 10.3389/frsle.2025.1593196 (PMC12435390; doi:10.3389/frsle.2025.1593196)
Supplement: Supplementary file 3 [file Data_Sheet_2.docx]

## **Supplementary Material: At-Home Kit Contents**

## **Study Kit Components Checklist**



**Electronics**


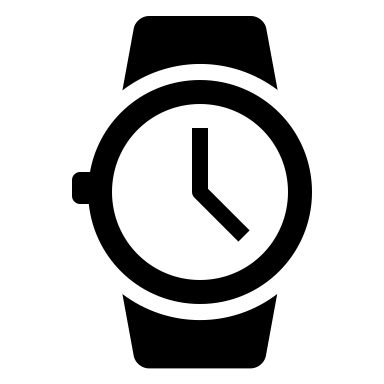


− ActTrust watch in a labeled envelope (1)

− Light Meter in a labeled envelope (1)
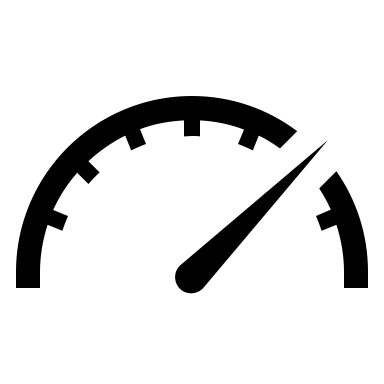


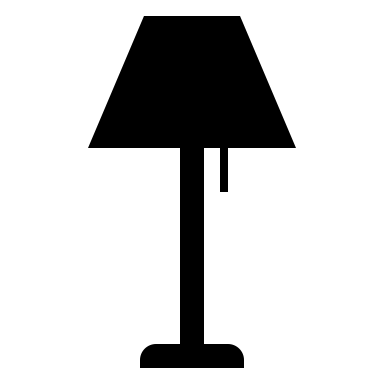


− Battery Operated Tea Lights (18)

−
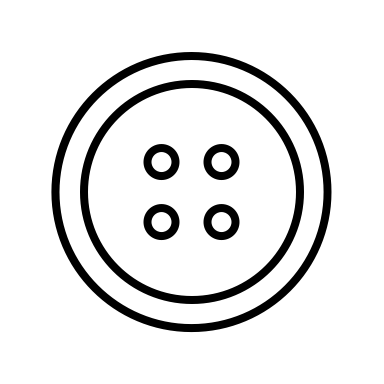
 iButton (1)

**Collection Tools**

- Clear bag labeled for DLMO collection containing the following:
  - Bottle with time stamp lid
  - 9 cotton collection swabs inside of the bottle with time stamp lid
  - 9 empty collection tubes
  - 12 labels, 9 for sample collection and 3 extra
  - 1 toothbrush
  - 1 Sharpie

**Other Study Materials**

 4 Black Trash Bags

 UVEX Blue light-blocking glasses

 Painters Tape

 2 freezer packs in resealable plastic pouch

 Clear bag with the following:

- Roll of medical tape
- 2 Tylenol single-use packs

**Shipping Materials**

 Silver envelope labeled for DLMO Collection

 Return shipping label

 Return shipping pouch

 Return shipping tape

 Return shipping box (same as kit box

- 2 extra collection tubes with

cotton swabs
