# Supplementary material for: Feasibility and acceptability of self-directed, remote dim-light melatonin onset collection in pediatric patients diagnosed with chronic pain
Source: Front Sleep. 2025 Jul 10;4:1593196. doi: 10.3389/frsle.2025.1593196 (PMC12435390; doi:10.3389/frsle.2025.1593196)
Supplement: Supplementary file 4 [file Data_Sheet_3.docx]

**Supplemental Material: At-Home Kit Instructions**


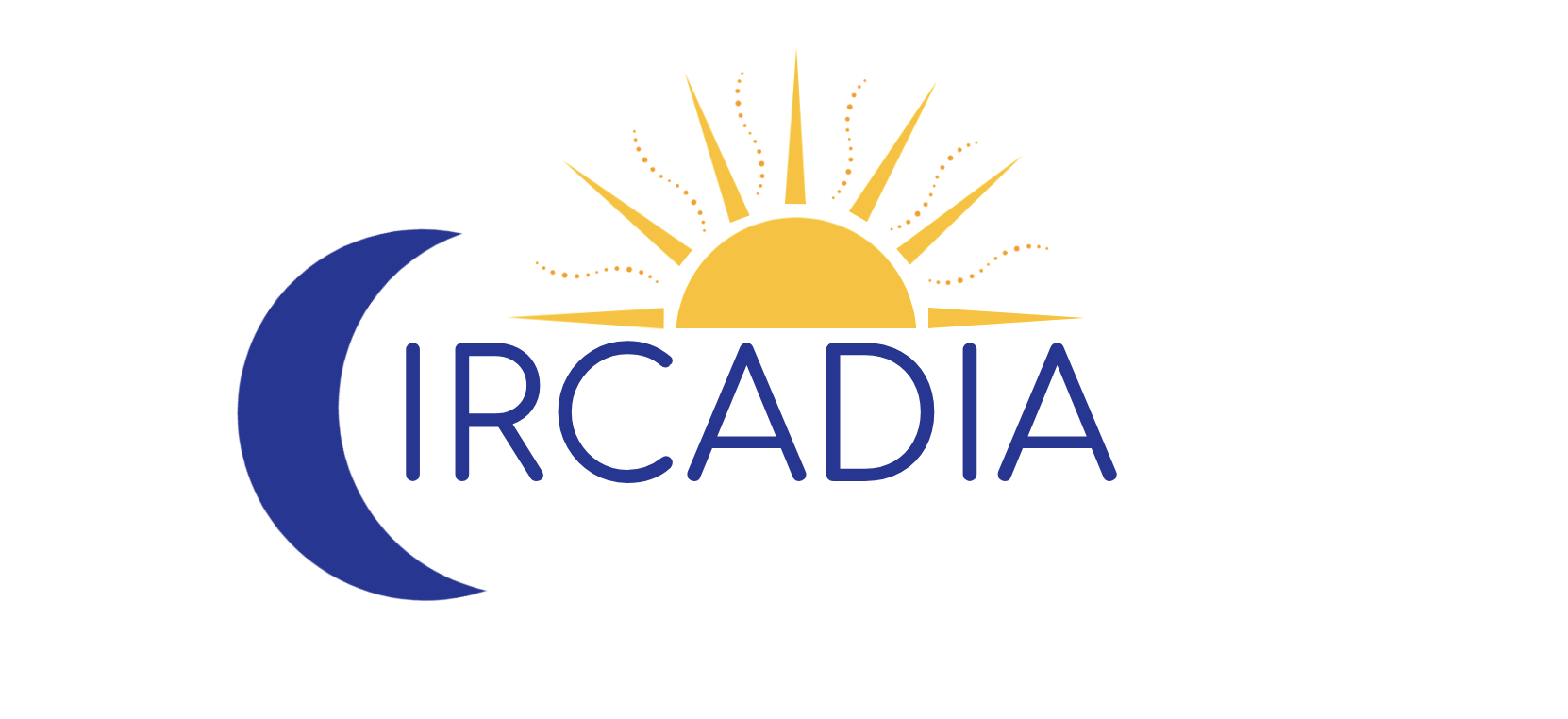


# **At-Home Study Kit Instructions**


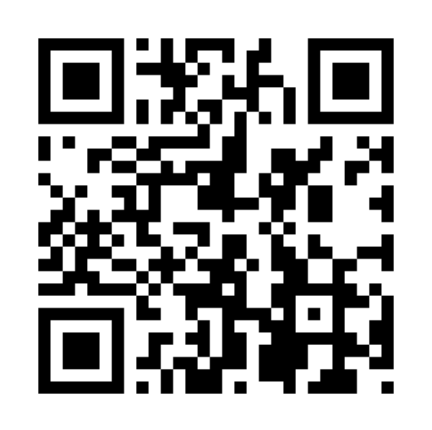


**Welcome! Thank you for participating in the Pediatric Circadia research study!**

We are a clinical research study located at Boston Children’s Hospital dedicated to better understanding the biomarkers of circadian rhythms in the pediatric population. Our study is self-directed and completely home-based, making study participation far reaching, flexible, and focused on your schedule.

Circadian rhythms are an integral part of our functioning, regulating and syncing our human behavior and physiology with the external environment and the 24-hour day. While research has been able to identify core components of our molecular circadian clock, much is still unknown about the physiological mechanisms driving our circadian rhythms.

Working together, we believe that we will be able to make the physiological pathways related to circadian rhythms clearer and identify the components directly influencing circadian rhythms in children and young adolescents.

If you have this letter and instruction packet in hand that means you have successfully received our at-home study kit. In this packet you will find details about the study and your study kit, including:

- Overview of the Circadia Study………………………………………………..page 3
- Checklist of your study kit components……………………………………….page 6
- Study Goal 1 instructions……………………………………………………….page 7
- Study Goal 2 instructions…………………………………………………......page 10
- DLMO Saliva Sample Collection ………………………………………….page 11-21
- Return shipping instructions……………………………………………….page 23-24
- Return kit components checklist…………………………………………..….page 25

Additional instructions can be found on the participant portal at circadiastudy.org. As always, please reach out to us with any questions, concerns, or thoughts.

Sincerely,

Joe Kossowsky, PhD MMSc.

Research Principal Investigator

Boston Children’s Hospital

Assistant Professor, Harvard Medical School

(617) 877-0014


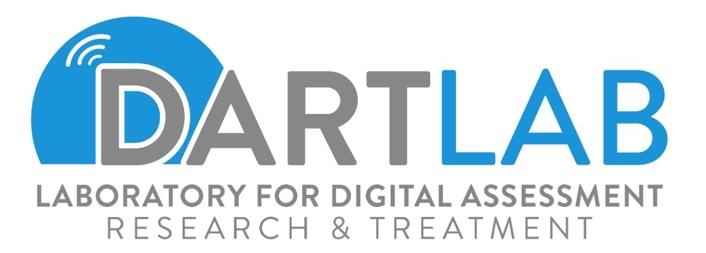


## **Overview of the Pediatric Circadia Study**



The Pediatric Circadia study is a 2-3 week study.

During the study you will complete:

- A Mini Sleep Log each day for 3 weeks
- One Dim-Light Melatonin Onset Collection (DLMO)
- 5 Questionnaires

See the study portal CircadiaStudy.org to complete the questionnaire and sleep logs.

This at-home kit is used for:

- One dim-light melatonin collection (DLMO)
- Activity tracking with the actigraphy watch

## **Collection Goals for the Circadia At-Home Study Kit**



This kit was created with you in mind, to help you easily and successfully provide your study collections. These include the following: activity and behavior monitoring and a measure of your melatonin levels provided through saliva samples collected hourly in dim light conditions. Below we explain the study Collection Goals:

**Goal 1)** **Complete Study Monitoring and Logging**

An important aspect of the Circadia study is better understanding the attributes of your daily activity, light exposure, and sleep and wake time. We will measure your activity and light exposure through a watch-like wearable called an **ActTrust.** We will assess your sleep behavior and mood using surveys.

**Goal 2) Provide hourly melatonin saliva samples in a dim light environment**

Melatonin is a hormone that helps regulate our sleep-wake cycle. You will schedule the one date for the melatonin sample collections, at least 5 days after study enrollment, based on your schedule. We have provided scheduling, preparation, and collection instructions on **pages 8-14**. To achieve this goal, you will use the following:

| Bottle with time stamp lid and cotton collection swabs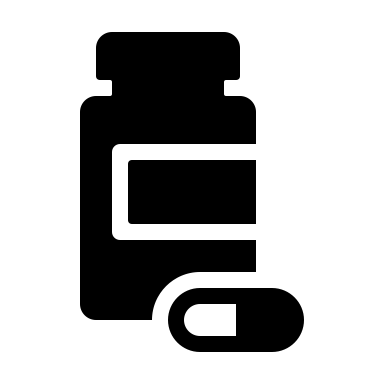 | Temperature monitor 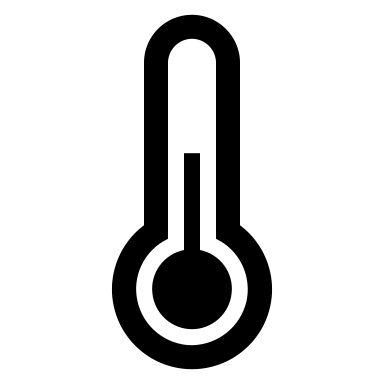 | *Misc. Items: UVEX Blue light-blocking glasses, toothbrush, tape, icepacks, insulated pouch.* |
| --- | --- | --- |
| 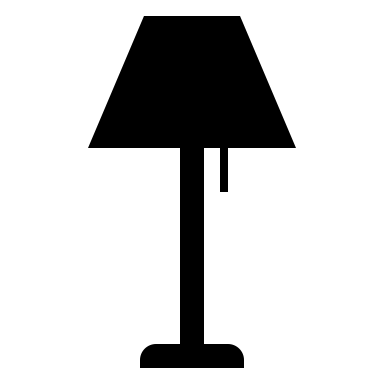  Night lights and light meter | 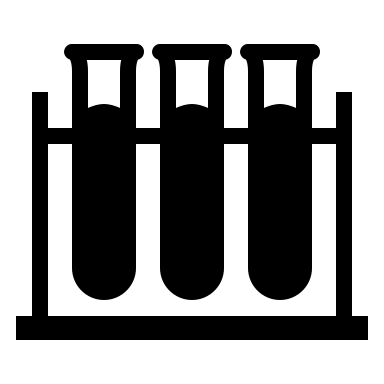  Collection Tubes |  |

|  |  |  |
| --- | --- | --- |

# **Study Checklists and Guides**


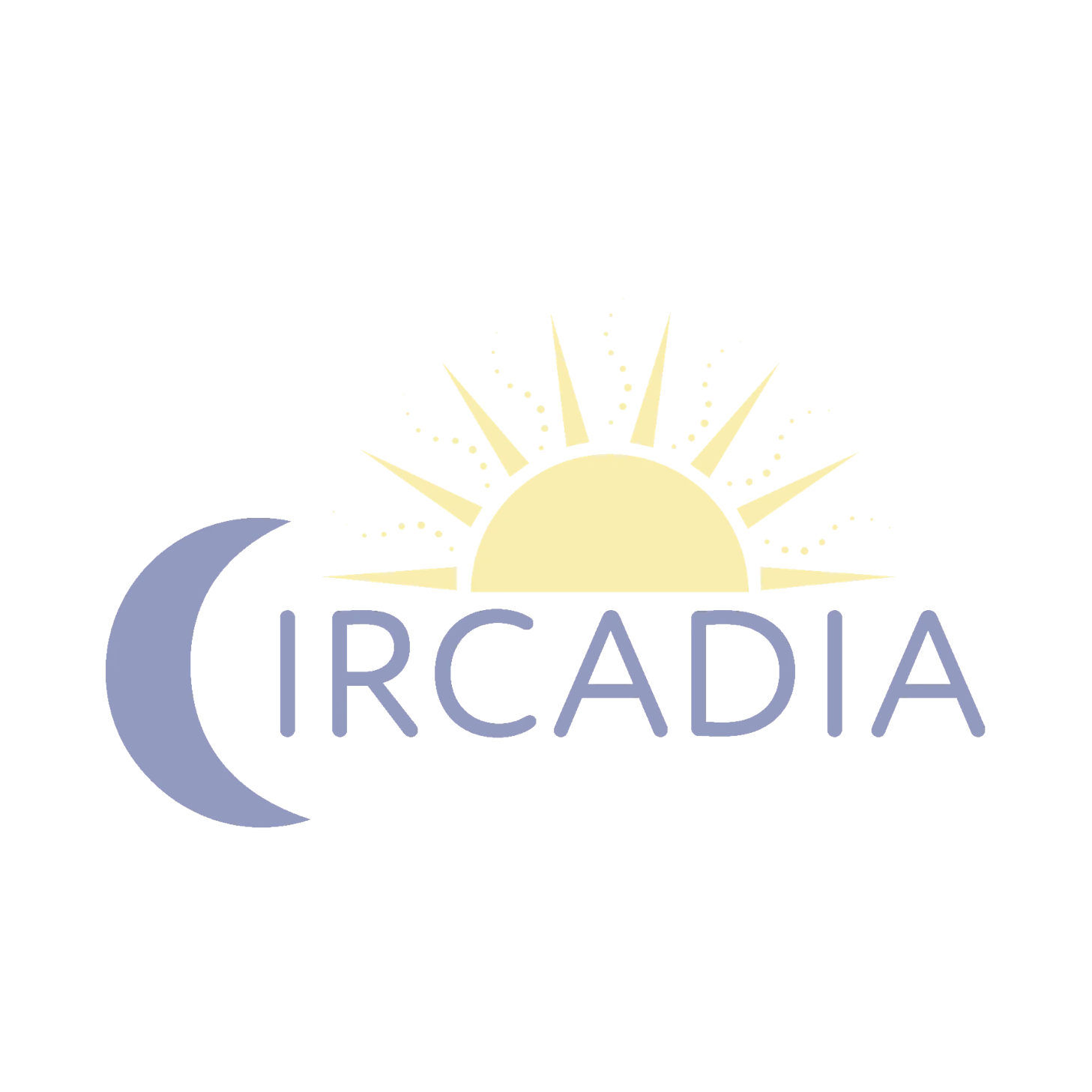


## **Study Kit Components Checklist**



**Electronics**


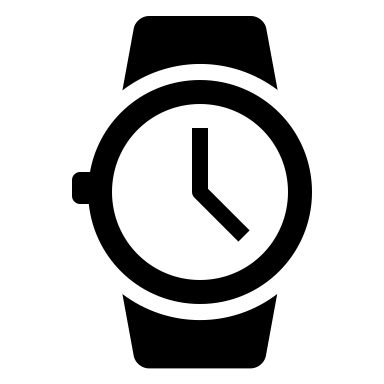


- ActTrust watch in a labeled envelope (1)

- Light Meter in a labeled envelope (1)
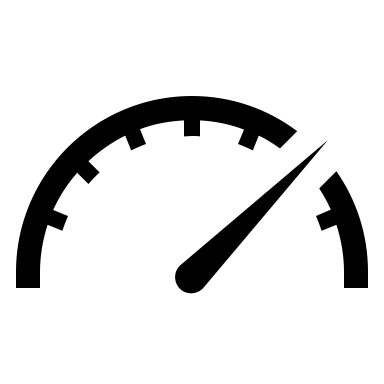


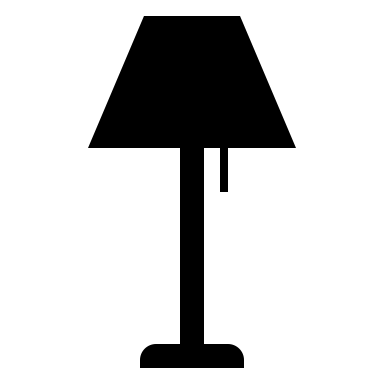


- Battery Operated Tea Lights (18)

-
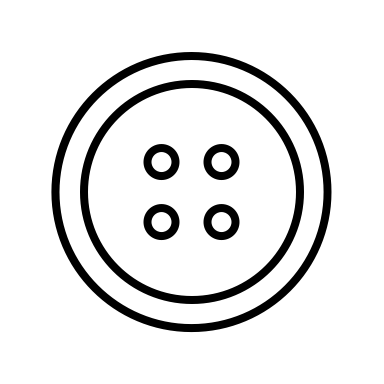
 iButton (1)

**Collection Tools**

- Clear bag labeled for DLMO collection containing the following:
  - Bottle with time stamp lid
  - 9 cotton collection swabs inside of the bottle with time stamp lid
  - 9 empty collection tubes
  - 12 labels, 9 for sample collection and 3 extra
  - 1 toothbrush
  - 1 Sharpie

**Other Study Materials**

 4 Black Trash Bags

 UVEX Blue light-blocking glasses

 Painters Tape

 2 freezer packs in resealable plastic pouch

 Clear bag with the following:

- Roll of medical tape
- 2 Tylenol single-use packs

**Shipping Materials**

 Silver envelope labeled for DLMO Collection

 Return shipping label

 Return shipping pouch

 Return shipping tape

 Return shipping box (same as kit box)

- 2 extra collection tubes with cotton swabs

# **Goal 1: Complete Study Monitoring using ActTrust and Mini Sleep Log**


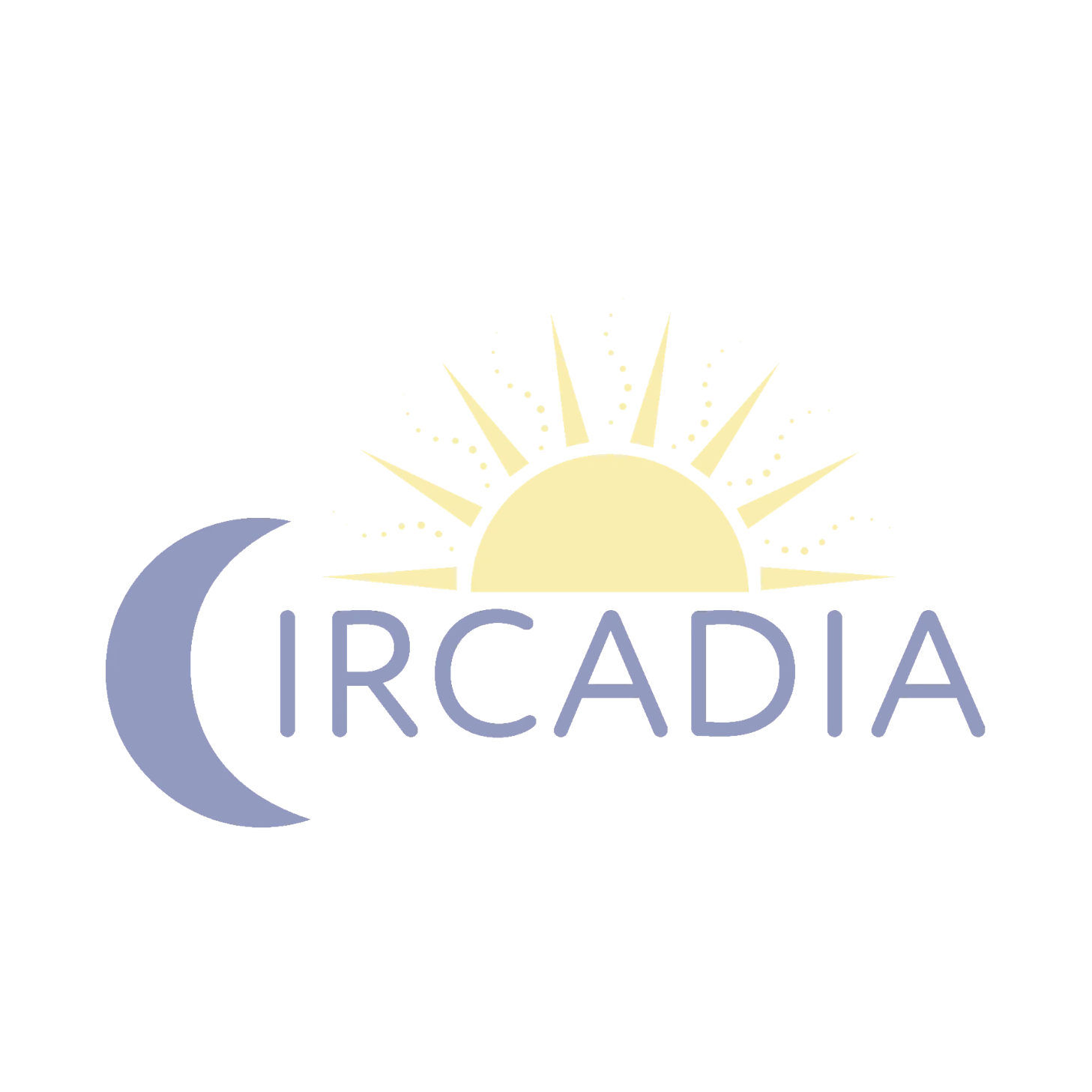


**Using the ActTrust**



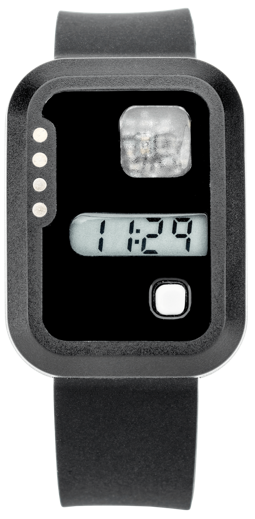


To use the ActTrust, unhook the clasp and place the band on your **non-dominant hand**.

For example, if you write with your right hand, you will wear this on your left hand for the duration of the study.

Adjust the strap then clasp it, ensuring a snug but comfortable fit. Ensure the face of the ActTrust is not covered by any clothing.

## **ActTrust Instructions**



- Wear the ActTrust for the duration of the at-home protocol. This will be for 24 hours a day for about 3 weeks.
- The ActTrust may be worn while showering or washing your hands. You may take the ActTrust off if you prefer to not get it wet. If you remove it, please put it on immediately after your activity.
- **To mark the start of your bedtime,** press the button on the ActTrust prior to going to sleep
- **To mark your waketime,** press the button on the ActTrust immediately upon waking
- If the ActTrust at any point causes skin sensitivity, use the provided medical tape to wear underneath the band. If the sensitivity persists, you may remove the ActTrust. Contact the study team if this occurs.

## **Mini Sleep Log**

Complete the Mini Sleep Log each day on the Patient Portal through StudyTrax.

## **Daily Study Checklist**

The following checklist is for daily use for the duration of the Circadia Study. Use the calendar to keep track of completed days.


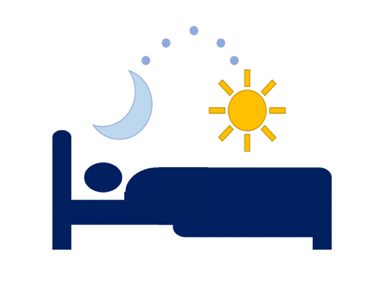


- **Complete Mini Sleep Log**

|  | Sunday | Monday | Tuesday | Wednesday | Thursday | Friday | Saturday |
| --- | --- | --- | --- | --- | --- | --- | --- |
| Week 1 | **○**Sleep Log | **○**Sleep Log | **○**Sleep Log | **○**Sleep Log | **○**Sleep Log | **○**Sleep Log | **○**Sleep Log |
| Week 2 | **○**Sleep Log | **○**Sleep Log | **○**Sleep Log | **○**Sleep Log | **○**Sleep Log | **○**Sleep Log | **○**Sleep Log |
| Week 3 | **○**Sleep Log | **○**Sleep Log | **○**Sleep Log | **○**Sleep Log | **○**Sleep Log | **○**Sleep Log | **○**Sleep Log |
| Week 4 | **○**Sleep Log | **○**Sleep Log | **○**Sleep Log | **○**Sleep Log | **○**Sleep Log | **○**Sleep Log | **○**Sleep Log |
| Week 5 | **○**Sleep Log | **○**Sleep Log | **○**Sleep Log | **○**Sleep Log | **○**Sleep Log | **○**Sleep Log | **○**Sleep Log |
| Week 6 | **○**Sleep Log | **○**Sleep Log | **○**Sleep Log | **○**Sleep Log | **○**Sleep Log | **○**Sleep Log | **○**Sleep Log |

# **Goal 2: Provide Hourly Melatonin Saliva Samples in a Dim Light Environment**


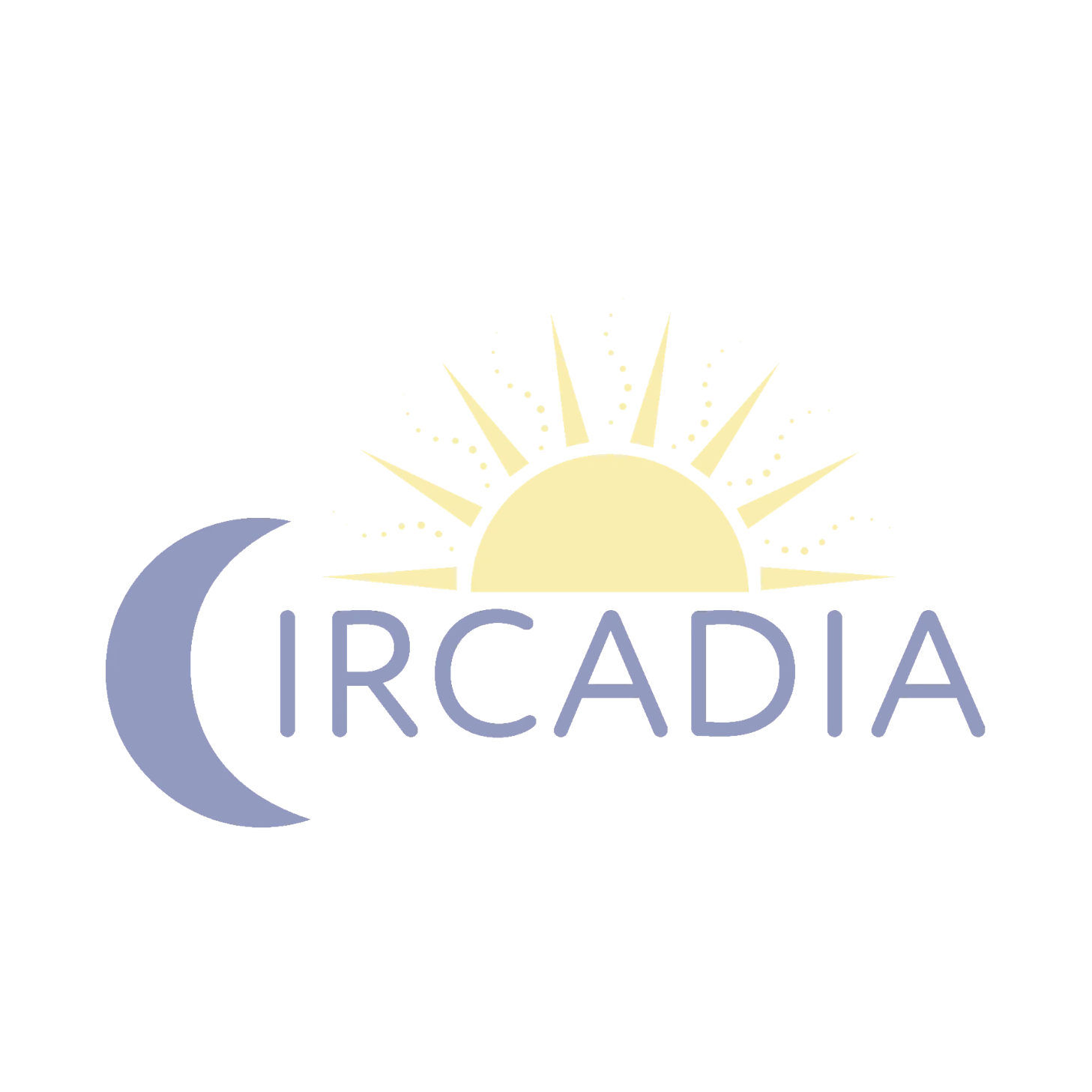


## **Dim Light Melatonin Onset (DLMO)**

## **Saliva Sample Collection**

**Attention: Icepacks must be frozen prior to start of sample collection**

**Overview:** The Dim Light Melatonin Saliva Sample Collection kit is designed to collect saliva samples over an 8-hour period overlapping your bedtime in one-hour intervals. Melatonin is a hormone that regulates your sleep-wake cycle. The melatonin concentration from your samples will be analyzed to provide us your biological bedtime.

**Melatonin levels are affected by light**. For the sample collection, it is extremely important to carefully follow instructions to ensure light is not affecting your samples. We will be tracking the saliva collection times. Please follow scheduled sample times and open and close the cotton swab bottle with the timestamp lid **fully** after each collection.

**Protocol Duration**: ~8 hours.


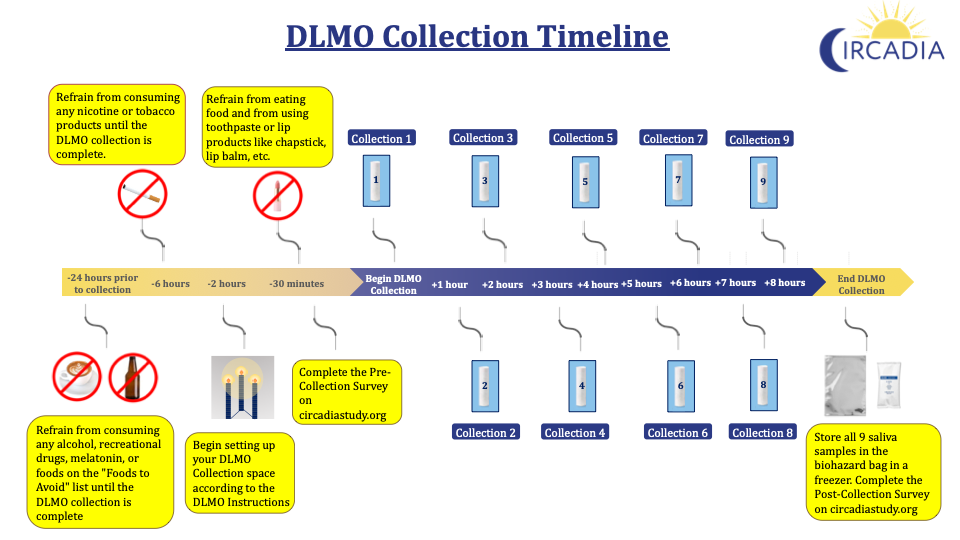


## **3 Steps for DLMO Sample Collection Success**



**1. Your food, drink, and medication:** Monitor your **food, drink, and medication** consumption for 24 hours prior to, and during the study.


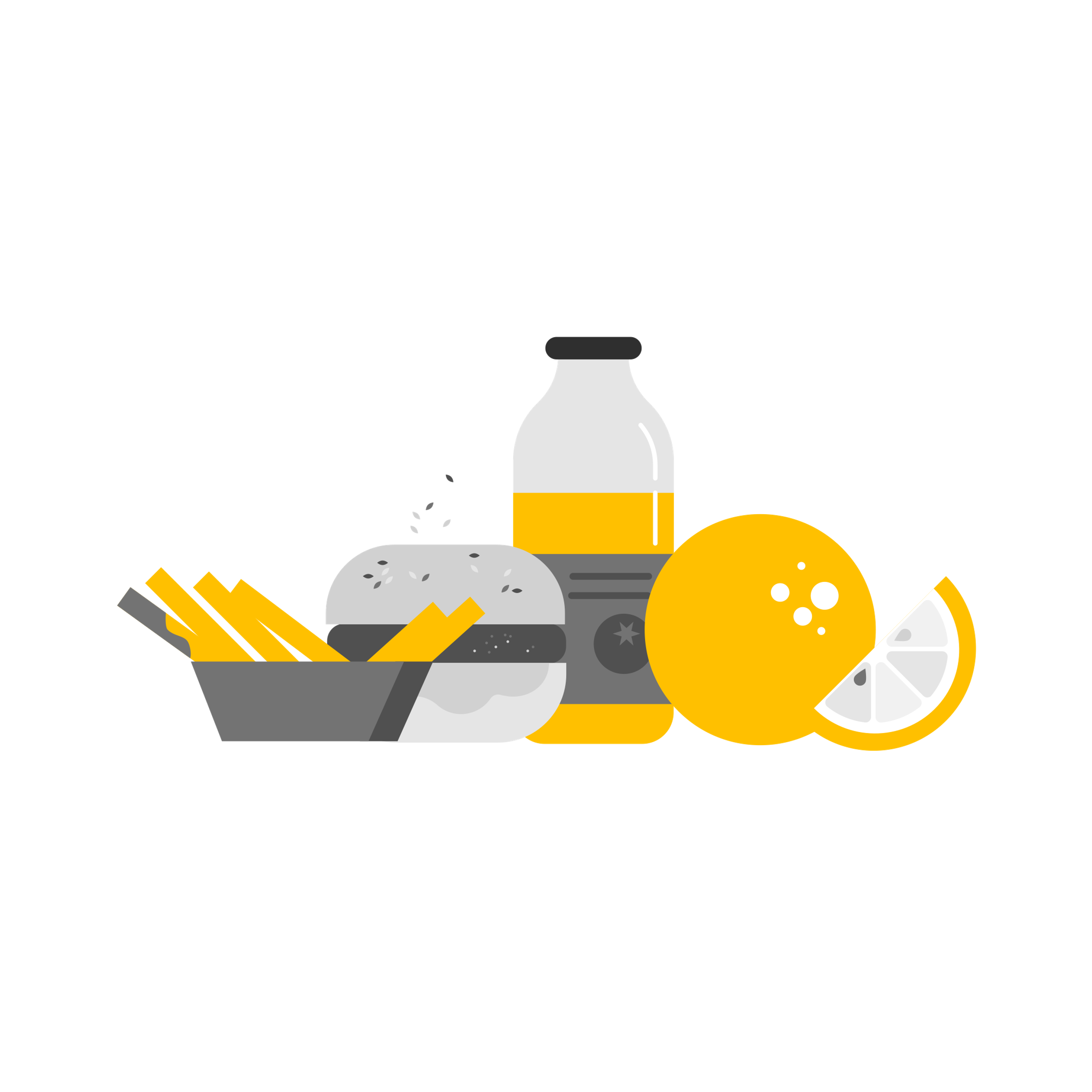


**2. Your room:** Keep your room dim and relaxed during the study.


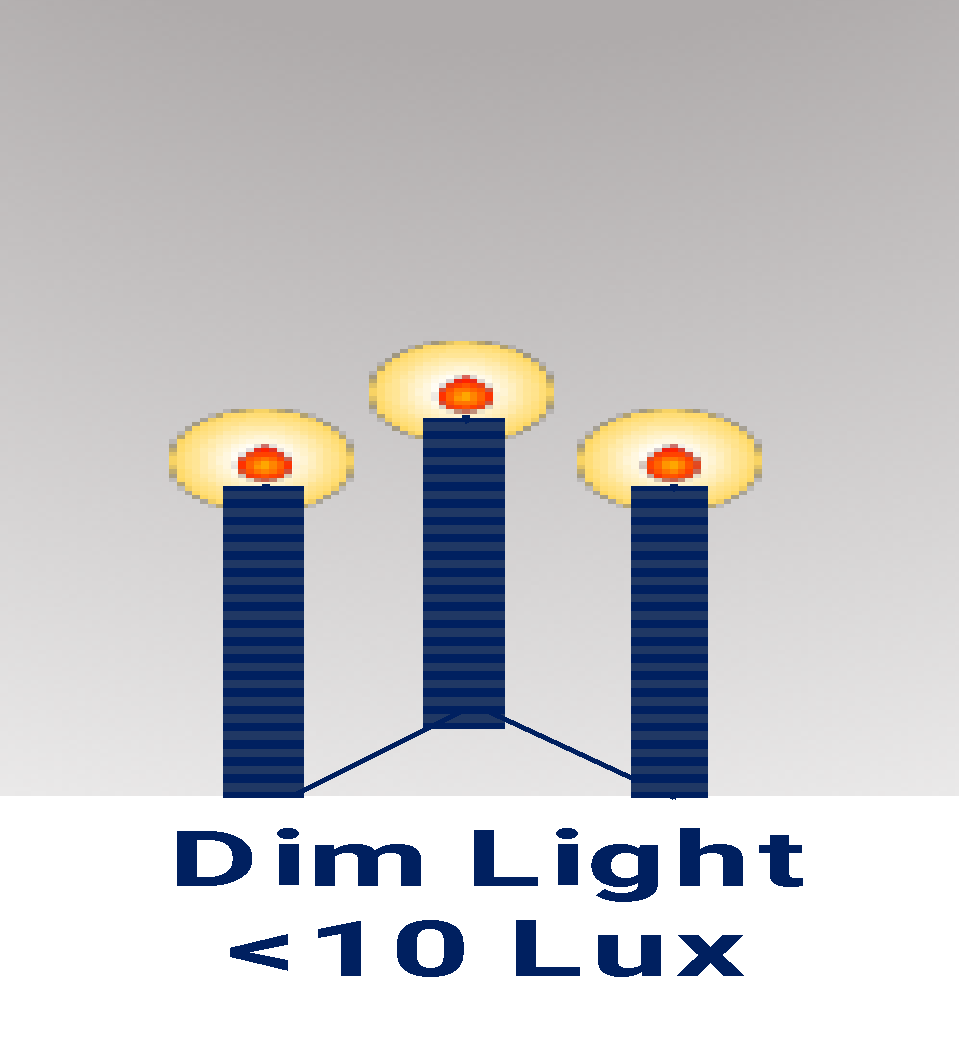


**3. Your saliva samples:** Keep samples free of food, collect them on time, and store them cold.


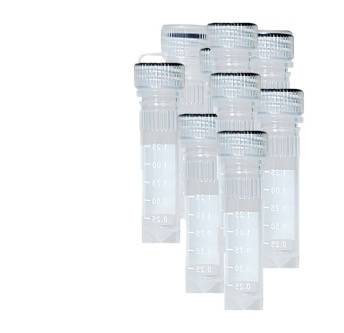


The following pages describe how to complete these steps.

**Step 1. Monitor your food, drink, and medications.**



| **24 Hours** Prior to Collection | 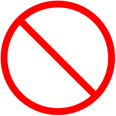  **No** alcohol, caffeine, or 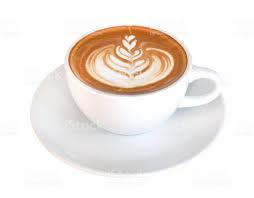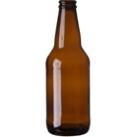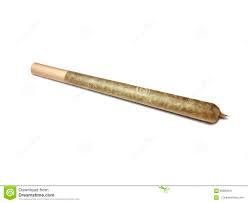  recreational drug consumption.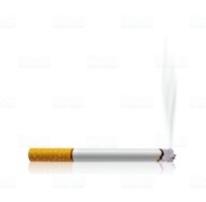  **No** nicotine or tobacco products  including but not limited to cigarettes,  vaping, chewing tobacco, pipes, 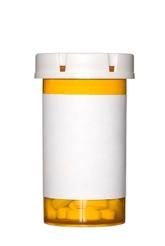  cigars.  **No** non-steroidal anti-inflammatory drugs (NSAIDs) or melatonin, if pain mediciation is  Needed please take provided Tylenol |
| --- | --- |
| **Day of Collection** | 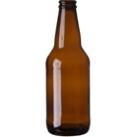  **No** alcohol, caffeine, or 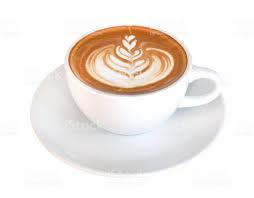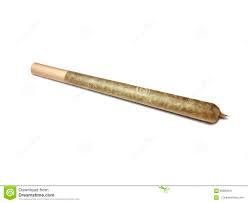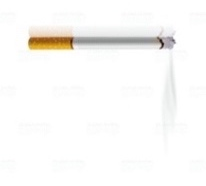  recreational drug consumption.  **No** nicotine or tobacco products  including but not limited to cigarettes,  vaping, chewing tobacco, pipes,  cigars.  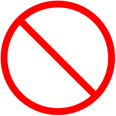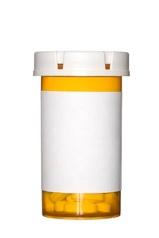  **No** non-steroidal anti-inflammatory drugs (NSAIDs) or melatonin, if pain mediciation is  Needed please take provided Tylenol  *Avoid the following:*  Food: Turkey, bananas, citric acid, 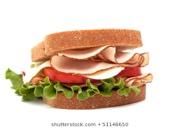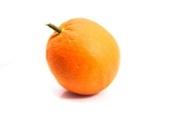  foods containing Red 40 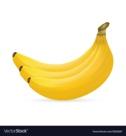  (see Foods to Avoid) |
| **6 Hours** Prior to Collection | **No** alcohol, caffeine, or  recreational drug consumption. 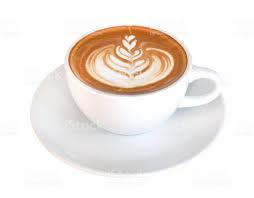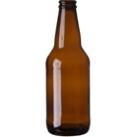  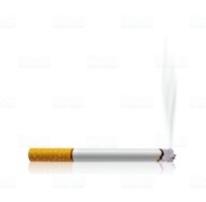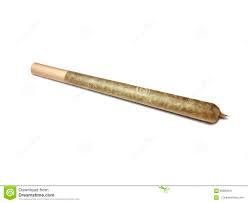  **No** nicotine or tobacco products  including but not limited to cigarettes,  vaping, chewing tobacco, pipes, 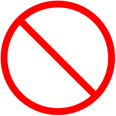  cigars.  **No** non-steroidal anti-inflammatory drugs (NSAIDs) or melatonin, if pain mediciation is  Needed please take provided Tylenol 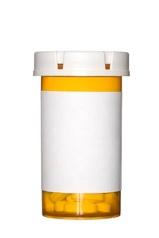  Do **NOT** consume the following: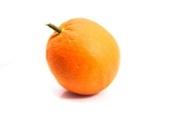  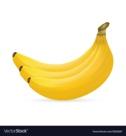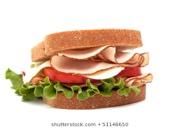  Drinks: Coffee, energy drinks,  caffeinated tea, caffeinated soda,  Food: Turkey, bananas, citric acid,  foods containing red 40, chocolate  (see Food Items to Avoid) |
| **Immediately before and during collection** | 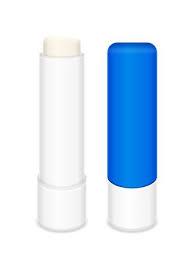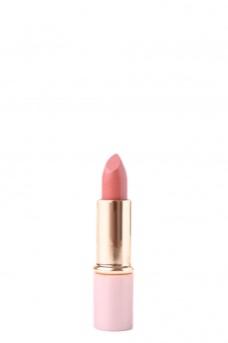 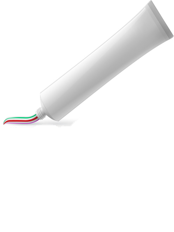 Toothpaste  **No** toothpaste when brushing teeth. 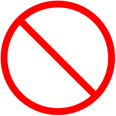  **No** lip products such as chapstick,  lip balm, or lipstick. |

**Melatonin Sample Collection: Food Items to Avoid**



The following is a list of food items to avoid when preparing for the melatonin sample collection. Melatonin levels can be affected by food; therefore, it is very important to avoid any food or drink that may influence your melatonin levels. The following list is for guidance but is not exhaustive. Contact the study team if you are unsure whether a snack or drink is compliant with the study protocol.

| **Food Containing Red 40** | Cereal  *Fruity Pebbles, Fruity Cheerios, Trix, Cap‘n Crunch Berry Flavor*  Food  *Yogurt, Jello, Condiments, Pickles, some cherry pie fillings*  Drinks  *Powerade Orange, Crush, Sunny Delight Orange Strawberry*  Candy  *M&Ms, Reese’s Pieces, Strawberry Twizzlers, Skittles, Peeps, Candy Corn, Jellybeans* | 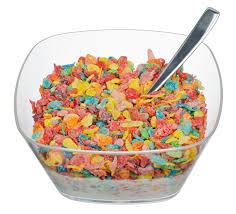  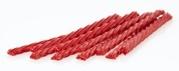  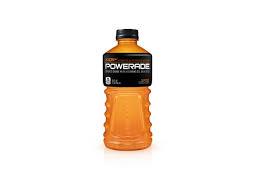  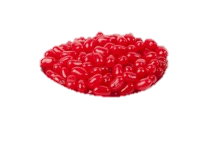  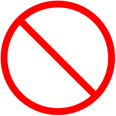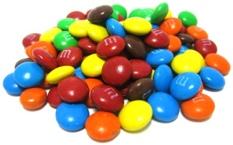 |
| --- | --- | --- |
| **Food Containing Citric Acid** | Drinks  *Orange Juice, Grapefruit Juice*  Fruits  *Lemons, limes, oranges, grapefruits, tangerines, pomelos, pineapple, strawberries, raspberries, cranberries, cherries, tomatoes*  Other  *Ketchup* | 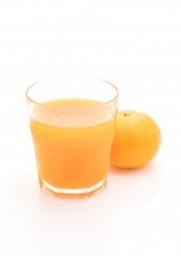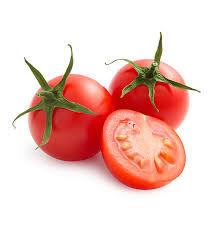  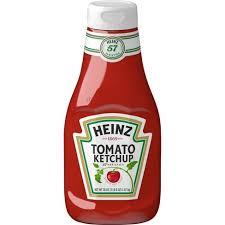  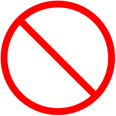  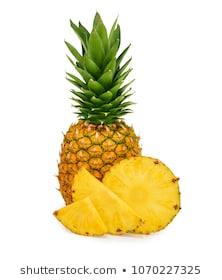 |
| **Food and Drink Containing Caffeine** | Drinks  *Coffee, Soda, Energy Drinks, Tea (Black, Green, White)*  Food  *Chocolate, Chocolate Flavored Cereal, Chocolate Pudding, Chocolate Cake, Ice Cream (Chocolate, Coffee flavors)*  Other  *Excedrin Pain Reliever* j | 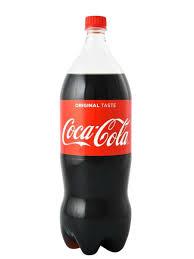  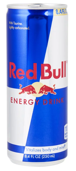  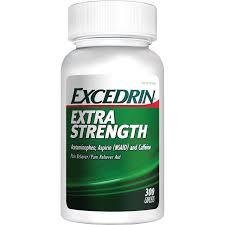  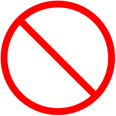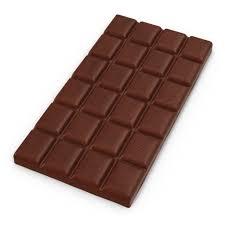 |
| **Food and Drink Containing Tryptophan** | Food  *Turkey, Tuna, Octopus, Crab, Tuna, Lamb Shoulder, Chicken breast, Goat, Ground pork, Pork tenderloin, Cheddar, Grated parmesan, Chia seeds, Pumpkin and Squash seeds, Sesame seeds* | 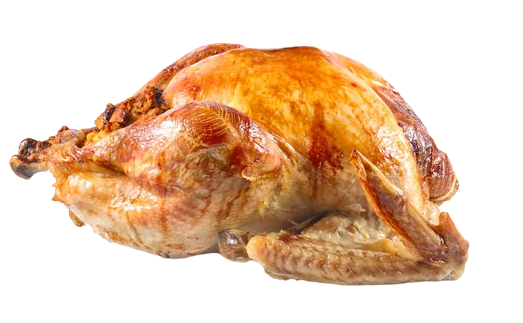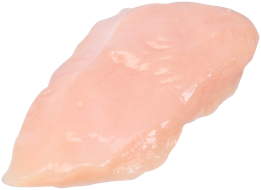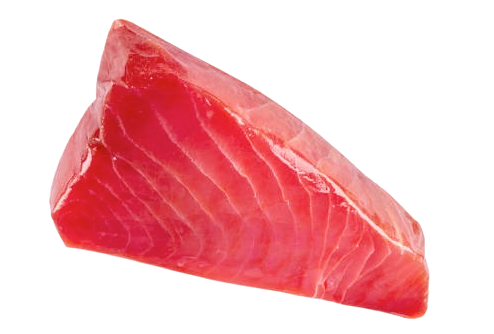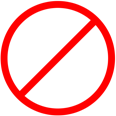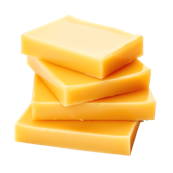 |

**Melatonin Sample Collection: Acceptable Food Options**



The following is a list of acceptable food options in case you are hungry and choose to eat during the sample collection. Melatonin levels can be affected by food; therefore, it is very important to choose snacks and drinks that are compliant with the study protocol. The following list is for guidance but is not exhaustive. Contact the study team if you are unsure whether a snack or drink is compliant with the study protocol.

| **Drinks** | Caffeine-free herbal teas  *Mint tea*  *Chamomile*  Caffeine-free sodas  *Root-beer*  Milk, Plain or Chocolate  *Cow Milk*  *Almond Milk*  *Soy Milk*  *Coconut Milk*  Flavored Seltzer or Sparkling Water | 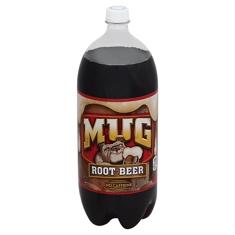  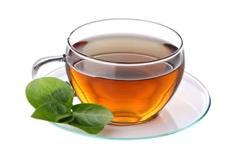  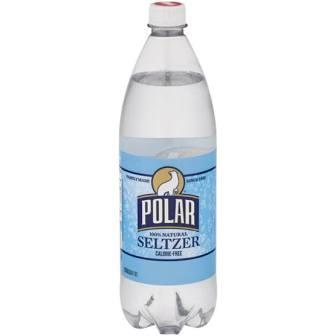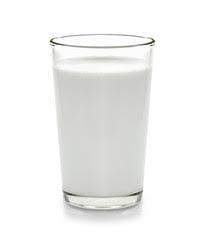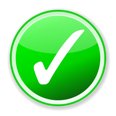 |
| --- | --- | --- |
| **Snacks** | Cup Snacks  *Apple sauce*  *Vanilla pudding*  Crackers  *Triscuits*  *Cheez-Its*  *Goldfish Crackers*  *Rice Thins (gluten-free)*  *Stauffer’s Animal Crackers*  *Graham Crackers*  Granola bars  *Kashi Granola Bars (Honey Almond Flavor)*  *Nature Valley Brand (Oats n Honey, Cinnamon, Peanut Butter)*  String Cheese  Light butter popcorn | 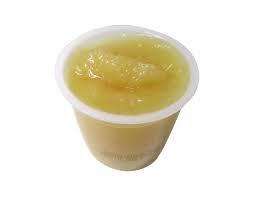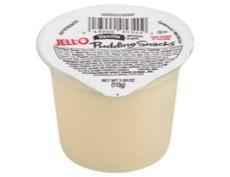  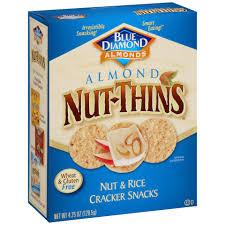  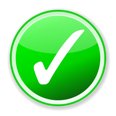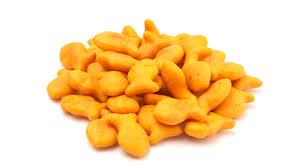  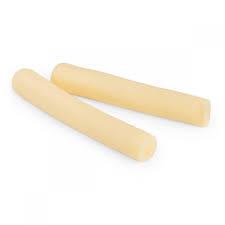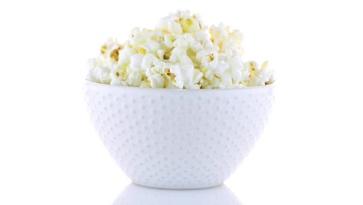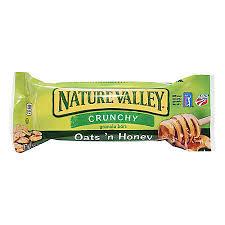 |
| **Fruits** | Apples  Pears  Watermelon  Blueberries  Cantaloupe  j | 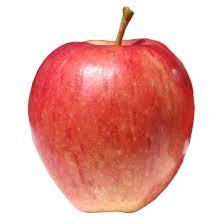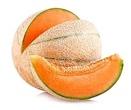  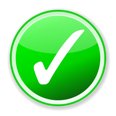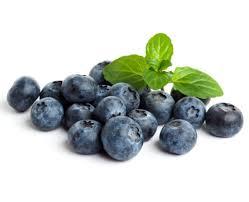 |

**Step 2. Keep your room dim and relaxed**



**Part 1: Preparing your Environment**

This protocol needs to be conducted in an area of rest, where you won’t be disturbed or exposed to bright light for the entirety of the collection period, about 8 hours. For this protocol a dim light environment below 10 lux must be maintained. A light meter and tea lights have been provided to facilitate maintaining dim light conditions. Blue light blocking glasses have been provided to minimize exposure to blue light. The following instructions will help you prepare your area of rest to ensure proper dim light conditions are maintained for the duration of the protocol:

1. Using the provided tape, tape down light switches in your collection space, nearest restroom, and any other areas where light may be needed during the protocol. You may need to tape the provided aluminum foil or black trash bags over windows to block light.
2. Place up to 16 of the provided battery-operated tea lights in your collection space and 2 battery-operated tea lights in your bathroom. This will ensure dim light is maintained if the bathroom is needed during the protocol.
3. The provided light meter is handheld. When you are ready to begin sample collections and you have set your environment to dim light conditions, use the light meter to measure your light exposure. Hold the light meter at eye level and eight inches from your body. The meter should read below 10 lux. Please text the value on the light meter to the study phone number.
4. When dim light conditions under 10 lux have been achieved, you are ready to begin the protocol.
5. You may only use electronics such as a TV or cell phone if they have been dimmed to the lowest setting. If using either of these devices, change their settings to the dimmest light setting before beginning the sample collection. You may use a laptop provided it is on the dimmest setting, with night mode activated if possible, and the laptop is AT LEAST 2 feet (24 inches) away from your face. Electronic tablets (for example iPads, Kindles) are not allowed. You must take these precautions the whole 8 hours.

**Melatonin Sample Collection: Guidance on Light Intensity**



The diagram below depicts different light settings and their approximate lux. Lux is a measure of light intensity, equal to one lumen per square meter. That means that in a darkened room, one lux is approximately equal to the light emitted from one lit candle. Melatonin levels are diminished by higher lux levels; therefore, it is important for the melatonin sample collection to collect samples in dim light conditions.

**Internal Light Lux Measures**
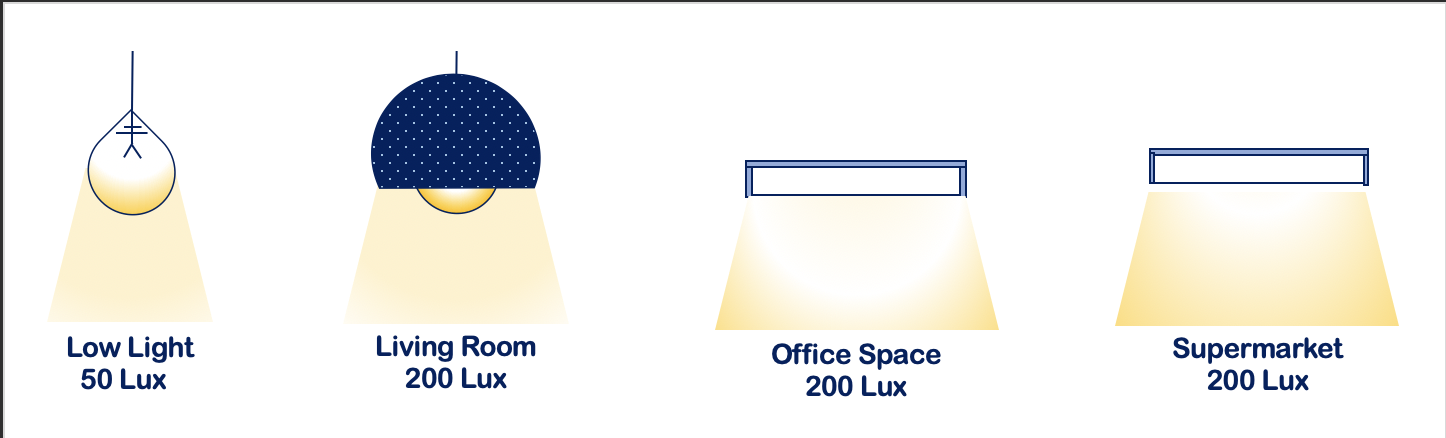


**External Light Lux Measures**
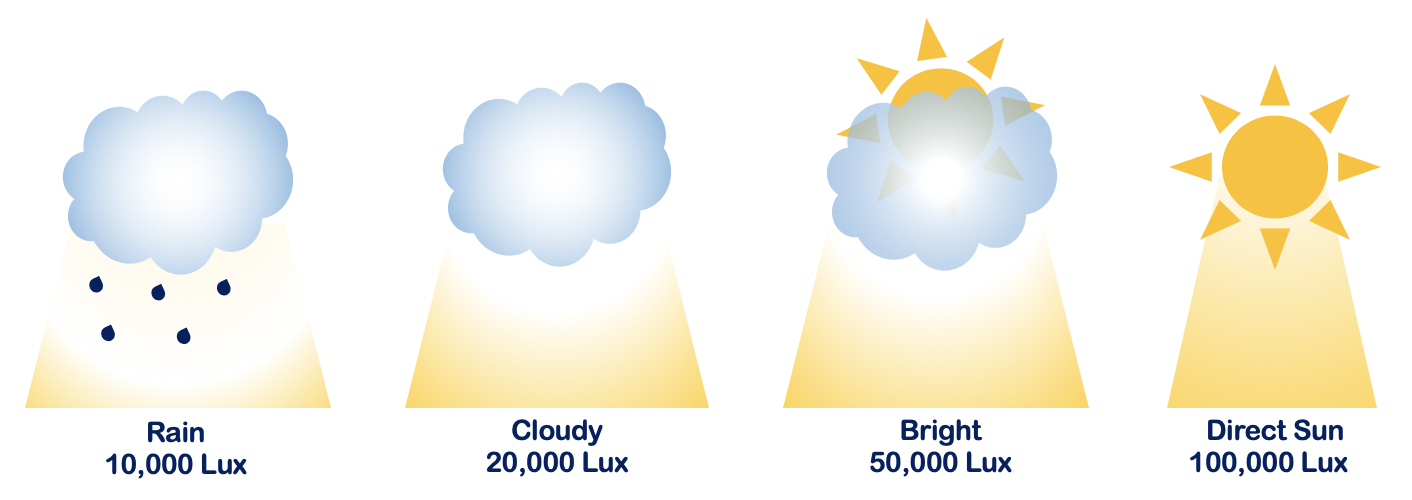


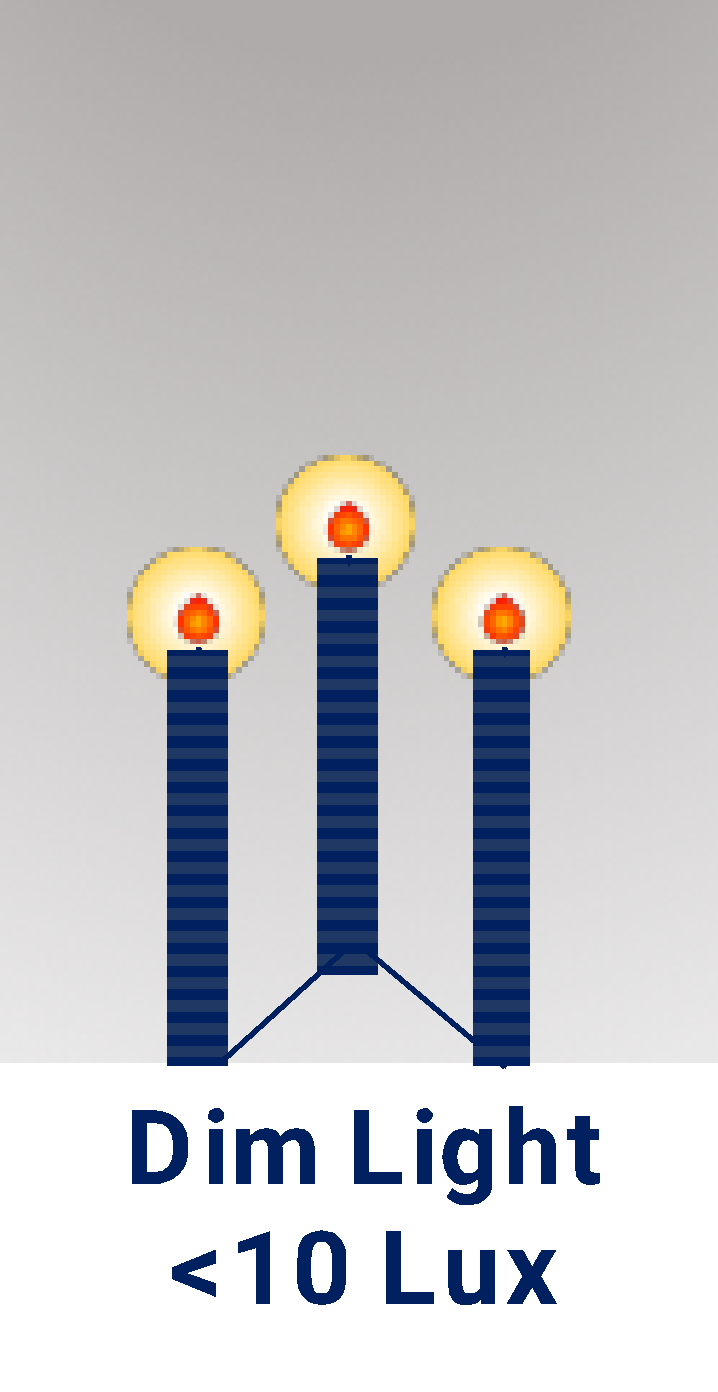


**For the melatonin sample collection, samples must be collected in dim light conditions.**

**Dim light conditions are defined as a collection space with light measuring below 10 lux.**

**Part 2: Preparing for Collection**

This protocol requires that you maintain a resting position for the duration of the protocol. The following instructions will prepare you to ensure resting conditions are maintained for the duration of the protocol:

1. You may place water and a snack near you in case you are thirsty or hungry during the protocol. Please see the provided guidance sheets for a list of study protocol appropriate food. *You may drink water or eat immediately* **AFTER** *a saliva sample collection, giving at least 30 minutes prior to your next collection and using a wet toothbrush with NO toothpaste to clean mouth and teeth.*
2. One bag of collection tubes has been provided; each bag contains 9 tubes. There are three spare tubes that you can use if you need to redo one. Take the bag of tubes and place the bag near you where tubes can be accessed easily.
3. You will label tubes with numbers 1-9 as you collect each sample every hour during the protocol. Write only the number directly on the side of the corresponding tube using the sharpie provided (e.g. first sample = 1).
4. Collected saliva samples will need to remain cold during the protocol. Ice packs and silver envelopes were provided with the collection kit. Place two frozen ice packs into the silver envelope and place it near you, where it can be accessed easily.

***Example Room Set Up***

### **Step 3. Collect and store your saliva samples.**

1. Open the white bottle with time stamp lid at the scheduled sample collection time and remove **one** cotton collection swab then replace the cap and twist it completely closed until the two black lines on the side align. The cap will automatically track the time the bottle was opened and closed.
2. Place the cotton collection swab in your mouth and chew on it for 3-4 minutes to stimulate saliva production.
3. Once the cotton is fully saturated, remove it from your mouth and ensure to place the sample in the top part of the collection tube. The collection tubes have lids that are labeled 1-9, corresponding with each sample.
4. Using the provided marker, additionally label the side of the tube with the number of your sample, for example: 1 for Sample 1. This helps us ensure you used the correct tube for each sample.
5. Securely fasten the cap onto the tube. Tape the iButton on top of the **Sample 1** cap using the roll of tape included in the kit.
6. Place the completed sample into the biohazard bag, then place and keep the bag inside of the silver pouch with the ice pack.
7. Repeat steps 1-6 every hour on the hour until all nine samples are collected. At the end of the protocol, you should have nine samples labeled “1, 2, 3, 4, etc…”.
8. Place completed samples into the biohazard bag. Place that bag into the silver pouch containing a frozen icepack. Continue adding collected samples until all nine samples are completed. Place the silver pouch containing the icepack and biohazard bag with samples into your freezer.

#

# **Study Completion**

## **Study Completion: Returning Samples and Electronics**

The following instructions are provided to ensure collected samples are shipped appropriately. Icepacks should be fully frozen and remain in the silver envelope with collected samples. Use the cardboard box and foam liner to return ship study samples and material. Please **ONLY** package samples on the same day as planned return shipping.

| **Melatonin Collection Samples**  **.**  When ready to ship, place the silver envelope for DLMO Collection 1, containing frozen samples and frozen icepacks, into the foam box lining the cardboard shipping box. | |
| --- | --- |
| Shipping Temperature: **Frozen** |  |

| **Electronics**  **.**  Place the Light Meter and ActTrust in their original envelopes. Place these envelopes with electronics in the foam box lining the cardboard shipping box. | |
| --- | --- |
| Shipping Temperature: **Room Temperature** |  |

*Refer to the Return Shipping: Item Checklist to ensure all study material is returned.*

## **Study Completion: Shipping Samples and Electronics**

*The following instructions are provided to ensure collected samples are shipped appropriately. Please package samples on the same day as shipping.*

1. Once samples and electronics are in their respective insulated mailers and inside the foam box, close the foam box using the foam lid. Then seal the cardboard box using the provided shipping tape.
2. Place the provided FedEx shipping slip into the FedEx pouch as shown below. Remove the sticker from the back of the FedEx pouch and adhere it to the top of the sealed box.

1. Bring the kit to your nearest FedEx center. (You can locate Fedex centers near you online at <https://www.fedex.com/locate/>.) If you need help finding your nearest FedEx center, please reach out to the study team and we will happily assist you. The FedEx center will scan the kit and mail it to our study team **at no cost to you.**
2. In the participant portal, navigate to the “Kit Return” task and fill it out.

## **Return Shipping: Essential Items Checklist**

Collected Samples:

◎ Silver envelope labeled DLMO Collection 1 containing 9 sample tubes and an ice pack.

Electronics:

◎ ActTrust watch (1)

◎ Bottle with time stamp lid (1)

◎ Light Meter (1)

◎ Battery Operated Tea Lights (18)

◎ iButton (1)

Other:

◎ Blue Light-Blocking Glasses (1)

# **Thank you for participating**

# **in the Circadia Study!**
